# Deep Resequencing of GWAS Loci Identifies Rare Variants in *CARD9*, *IL23R* and *RNF186* That Are Associated with Ulcerative Colitis

**DOI:** 10.1371/journal.pgen.1003723

**Published:** 2013-09-12

**Authors:** Mélissa Beaudoin, Philippe Goyette, Gabrielle Boucher, Ken Sin Lo, Manuel A. Rivas, Christine Stevens, Azadeh Alikashani, Martin Ladouceur, David Ellinghaus, Leif Törkvist, Gautam Goel, Caroline Lagacé, Vito Annese, Alain Bitton, Jakob Begun, Steve R. Brant, Francesca Bresso, Judy H. Cho, Richard H. Duerr, Jonas Halfvarson, Dermot P. B. McGovern, Graham Radford-Smith, Stefan Schreiber, Philip L. Schumm, Yashoda Sharma, Mark S. Silverberg, Rinse K. Weersma, Quebec IBD Genetics Consortium, NIDDK IBD Genetics Consortium, International IBD Genetics Consortium, Mauro D'Amato, Severine Vermeire, Andre Franke, Guillaume Lettre, Ramnik J. Xavier, Mark J. Daly, John D. Rioux

**Affiliations:** 1 Montreal Heart Institute, Research Center, Montreal, Quebec, Canada; 2 Center for the Study of IBD (CSIBD) Genetics, The Broad Institute, Cambridge, Massachusetts, United States of America; 3 Institute of Clinical Molecular Biology, Christian-Albrechts-University, Kiel, Germany; 4 Department of Clinical Science Intervention and Technology, Karolinska Institutet, Stockholm, Sweden; 5 Center for Computational and Integrative Biology and Gastrointestinal Unit, Massachusetts General Hospital, Harvard School of Medicine, Boston, Massachusetts, United States of America; 6 Unit of Gastroenterology, Istituto di Ricovero e Cura a Carattere Scientifico-Casa Sollievo della Sofferenza (IRCCS-CSS) Hospital, San Giovanni Rotondo, Italy; 7 Azienda Ospedaliero Universitaria (AOU) Careggi, Unit of Gastroenterology SOD2, Florence, Italy; 8 Division of Gastroenterology, McGill University Health Centre, Royal Victoria Hospital, Montréal, Québec, Canada; 9 Meyerhoff Inflammatory Bowel Diseases Center, Department of Medicine, Johns Hopkins University School of Medicine, and Department of Epidemiology, Bloomberg School of Public Health, Johns Hopkins University, Baltimore, Maryland, United States of America; 10 Department of Medicine of the Karolinska University Hospital, Solna, Sweden; 11 Departments of Medicine and Genetics, Yale University, New Haven, Connecticut, United States of America; 12 Division of Gastroenterology, Hepatology and Nutrition, Department of Medicine, University of Pittsburgh School of Medicine, and Department of Human Genetics, University of Pittsburgh, Graduate School of Public Health, Pittsburgh, Pennsylvania, United States of America; 13 Department of Internal Medicine, Division of Gastroenterology, Örebro University Hospital and School of Health and Medical Sciences, Örebro University, Örebro, Sweden; 14 Cedars-Sinai F.Widjaja Inflammatory Bowel and Immunobiology Research Institute, and Medical Genetics Institute, Cedars-Sinai Medical Center, Los Angeles, California, United States of America; 15 Inflammatory Bowel Diseases, Genetic and Computational Biology, Queensland Institute of Medical Research, and Department of Gastroenterology, Royal Brisbane and Womens Hospital, and School of Medicine, University of Queensland, Brisbane, Australia; 16 Department for General Internal Medicine, Christian-Albrechts-University, Kiel, Germany; 17 Department of Health Studies, University of Chicago, Chicago, Illinois, United States of America; 18 Mount Sinai Hospital Inflammatory Bowel Disease Centre, University of Toronto, Toronto, Ontario, Canada; 19 Department of Gastroenterology and Hepatology, University of Groningen and University Medical Center Groningen, Groningen, The Netherlands; 20 Department of Biosciences and Nutrition, Karolinska Institutet, Stockholm, Sweden; 21 Division of Gastroenterology, University Hospital Gasthuisberg, Leuven, Belgium; 22 Université de Montréal, Faculté de Médecine, Montréal, Québec, Canada; 23 Broad Institute of MIT and Harvard University, Cambridge, Massachusetts, United States of America; 24 Analytic and Translational Genetics Unit, Massachusetts General Hospital, Harvard Medical School, Boston, Massachusetts, United States of America; Georgia Institute of Technology, United States of America

## Abstract

Genome-wide association studies and follow-up meta-analyses in Crohn's disease (CD) and ulcerative colitis (UC) have recently identified 163 disease-associated loci that meet genome-wide significance for these two inflammatory bowel diseases (IBD). These discoveries have already had a tremendous impact on our understanding of the genetic architecture of these diseases and have directed functional studies that have revealed some of the biological functions that are important to IBD (e.g. autophagy). Nonetheless, these loci can only explain a small proportion of disease variance (∼14% in CD and 7.5% in UC), suggesting that not only are additional loci to be found but that the known loci may contain high effect rare risk variants that have gone undetected by GWAS. To test this, we have used a targeted sequencing approach in 200 UC cases and 150 healthy controls (HC), all of French Canadian descent, to study 55 genes in regions associated with UC. We performed follow-up genotyping of 42 rare non-synonymous variants in independent case-control cohorts (totaling 14,435 UC cases and 20,204 HC). Our results confirmed significant association to rare non-synonymous coding variants in both *IL23R* and *CARD9*, previously identified from sequencing of CD loci, as well as identified a novel association in *RNF186*. With the exception of *CARD9* (OR = 0.39), the rare non-synonymous variants identified were of moderate effect (OR = 1.49 for *RNF186* and OR = 0.79 for *IL23R*). *RNF186* encodes a protein with a RING domain having predicted E3 ubiquitin-protein ligase activity and two transmembrane domains. Importantly, the disease-coding variant is located in the ubiquitin ligase domain. Finally, our results suggest that rare variants in genes identified by genome-wide association in UC are unlikely to contribute significantly to the overall variance for the disease. Rather, these are expected to help focus functional studies of the corresponding disease loci.

## Author Summary

Genetic studies of common diseases have seen tremendous progress in the last half-decade primarily due to recent technologies that enable a systematic examination of genetic markers across the entire genome in large numbers of patients and healthy controls. The studies, while identifying genomic regions that influence a person's risk for developing disease, often do not pinpoint the actual gene or gene variants that account for this risk (called a causal gene/variant). A prime example of this can be seen with the 163 genetic risk factors that have recently been associated with the chronic inflammatory bowel diseases known as Crohn's disease and ulcerative colitis. For less than a handful of these 163 is the causative change in the genetic code known. The current study used an approach to directly look at the genetic code for a subset of these and identified a causative change in the genetic code for eight risk factors for ulcerative colitis. This finding is particularly important because it directs biological studies to understand the mechanisms that lead to this chronic life-long inflammatory disease.

## Introduction

Inflammatory bowel diseases (IBDs) are classified as chronic relapsing inflammatory diseases of the gastrointestinal tract. The two major forms of IBDs are Crohn's disease (CD, OMIM 266600) and ulcerative colitis (UC, OMIM 191390). Both genetic and environment factors play a central role in the pathogenesis of the inflammatory response of IBDs [1].

Recent genome-wide association (GWA) studies and meta-analyses in IBD have shown great success, with the identification of 163 independent IBD risk loci. While some loci were shown to be specific to either CD or UC risk, most have been shown to impact on both diseases, supporting earlier claims that these diseases share genetic risk factors [2]. These recent studies have identified important disease pathways but the common SNPs identified, with generally modest effects, explain only 14% and 7.5% of disease variance for CD and UC, respectively [3].

Due to linkage disequilibrium in the genome and limitations of GWAS chip designs to date, genome-wide scans typically identify common variants that tag regions of variable sizes containing multiple candidate genes for disease susceptibility. Although there have been a few notable exceptions, most of the common associated SNPs do not clearly identify causal variants, and further studies are needed to highlight the causal gene in many associated regions [4]–[6]. Sequencing of exons within associated regions in order to identify rare variants with strong effect on disease has been proposed as a means to help identify the causal genes and to help explain a further portion of disease variance. We have recently performed a pooled next-generation sequencing study in Crohn's disease, and identified association to novel low-frequency and rare protein altering variants in *NOD2*, *IL23R*, and *CARD9*, as well as *IL18RAP*, *CUL2*, *C1orf106*, *PTPN22* and *MUC19* [7]. We opted to use a similar targeted pooled next-generation sequencing approach to study UC-associated regions from our recent meta-analysis of 3 independent genome-wide scans for UC [8]. Using this approach we identified putative causal variants significantly associated to UC in three of the 22 loci examined and identified variants of interest for an additional six loci.

## Results

### Sequence analyses

We selected 200 ulcerative colitis cases and 150 healthy controls of French Canadian ancestry from among samples collected by the NIDDK IBD Genetics Consortium. Samples were pooled in batches of 50 cases or 50 controls and normalized in order for the DNA pool to reflect sample allele frequencies. We targeted 55 genes from 14 UC-associated regions, as well as 7 regions identified in CD showing nominal replication in our UC GWAS study and an additional candidate gene (*ECM1*) reported in recent literature [6], [8]–[10]. PCR amplification primers were successfully designed to capture a total of 508 amplicons for a total of 305 Kb or 70% of our original target sequences. Of these 508 PCR reactions, 472 (93%) successfully amplified in each of the 7 sample pools and we used these to construct libraries for high-throughput sequencing on an Illumina Genome Analyzer II. This sequencing yielded large amounts of high-quality data for each pool, that captured 99% of our amplified target regions (283 Kb total; 117 Kb exonic sequences) and achieved 1575× median coverage per pool (corresponding to 31.5× per sample).

We used the previously described variant calling method Syzygy, designed to accommodate pooled study designs, to identify rare and low-frequency single nucleotide variants in our pooled samples [7]. Syzygy detected 1590 high confidence variants in our target regions, including 309 coding region variants (189 missense, 114 synonymous, 2 nonsense and 4 essential splice junction variants) with 56% of these already reported in dbSNP version 132, a non-synonymous/synonymous ratio of 1.7 and a transition/transversion ratio of 2.38 (Table S1). These results are similar to those obtained from our recent re-sequencing study in CD, as well as those reported by the 1000 Genomes Project, and are indicative of a relatively high true-positive rate for our dataset. This was confirmed by genotyping the 350 discovery DNA samples for a random subset of 237 variants from the total of 1590 high quality variants (Table S2).

### Follow-up genotyping and association analyses

After removal of variants that did not validate, variants observed only once in our sequencing dataset (singletons) and variants from the MHC region, 84 non-synonymous coding variants (missense, non-sense and splicing variants), were used for subsequent analyses. Following removal of common variants (frequency >5%) and variants that did not design in our genotyping assays, we carried out follow-up genotyping for 42 of these variants. Genotyping was performed in 6 independent case-control cohorts totaling 7,292 UC cases and 8,018 HC (Table S3), and additional data was obtained for 7,143 UC cases and 12,186 HC from the International IBD Genetics Consortium (IIBDGC) Immunochip project for 14 of these variants [3].

Since our study focuses on infrequent and rare variants, we expect few non-reference alleles for these variants in each subcohort studied, which precludes the use of asymptotic statistics utilized in typical association studies of common variants. Also, given the low frequencies of the variants tested, population structure is likely to be a more substantial problem and thus requires a stratified analysis with strict population case-control matching. We used a previously described mega-analysis of rare variants (MARV) approach that provides a permutation-based estimate of significance, within each sub-cohort, and accommodates variable numbers of case-control samples in each independent population for single-marker analysis [7].

**Figure 1:**
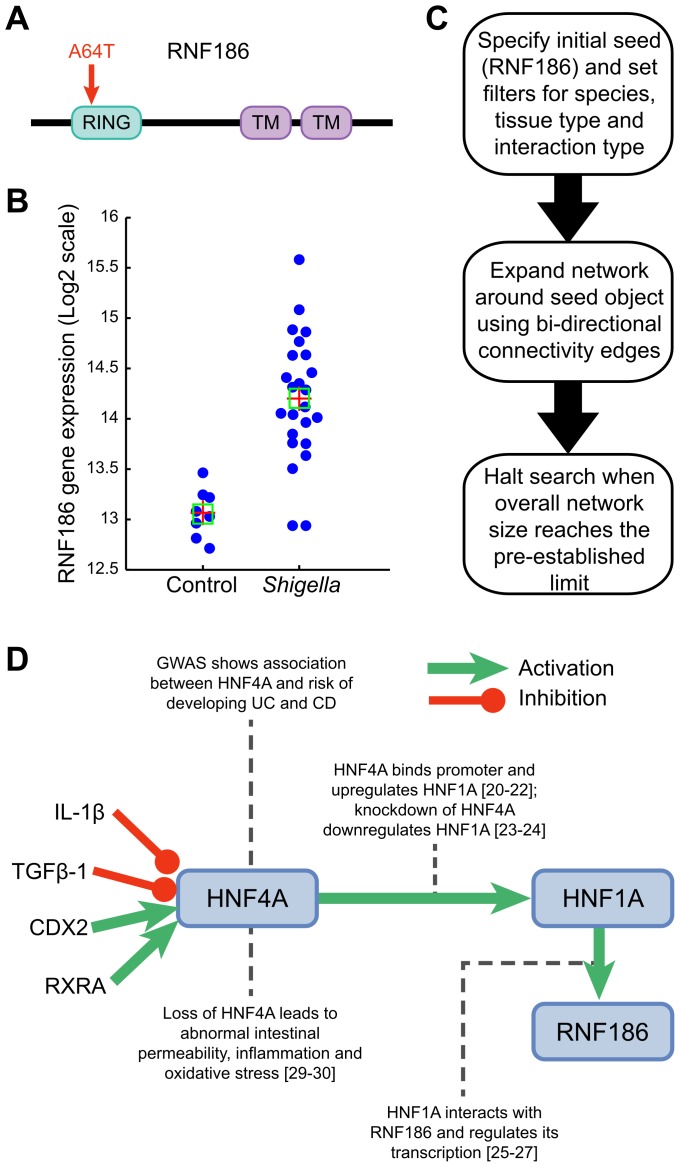
Functional characterization of RNF186. (A) *RNF186* encodes a protein with RING domain and two transmembrane domains. E3 ubiquitin-protein ligase activity is intrinsic to the RING domain. This domain contains the disease-coding variant (A64T). (B) *RNF186* expression response to *S. flexneri* in young mice (see also Figure S11). (C) Network building steps. Network is generated by mining multiple sources of interaction databases in Metacore that span human protein-protein, protein-DNA, Protein-RNA and protein-compounds interactions. (D) Transcriptional regulation model for *RNF186*. IL1-beta and TGF-beta 1 decrease *HNF4A* mRNA expression [39]–[41]. Knockdown of retinoid X receptor, alpha (*RXRA*) down-regulates *HNF4A* gene expression; RXRA interacts with *HNF4A* gene [24]. *HNF4A* is a direct target gene of caudal type homeobox 2 (CDX2); CDX2 increases *HNF4A* mRNA expression in intestinal epithelial cells [42], [43]. HNF4A binds promoter region of *HNF1A* and up-regulates its expression. HNF1A interacts with *RNF186* and regulates its transcription.

With a target set of 42 variants we can define a traditional corrected significance level of *P* = 0.0012 for our study. Three variants, located in the *CARD9*, *IL23R* and *RNF186* genes, reach this significance threshold suggesting that these could possibly be the causal genes/variants within these two loci (Table 1). Specifically, our results show that the c.IVS11+1G>C *CARD9* splice variant confers significant protection to UC (*P* = 1.47×10^−11^; OR = 0.39 [0.30–0.53]). We previously identified this splice variant in a sequencing project of CD loci and demonstrated that it leads to an alternatively spliced transcript that is missing exon 11 [7]. Our results also identify significant association to the valine to isoleucine substitution at position 362 (Val362Ile) in *IL23R* (*P* = 1.18×10^−03^; OR = 0.79 [0.68–0.91]) previously reported by a recent re-sequencing of positional candidates in Crohn's disease [7], [11]. The significantly associated rare variant that we identified in *RNF186* (*P* = 8.69×10^−4^; OR = 1.49 [1.17–1.90]) encodes an alanine to threonine substitution at position 64 (Ala64Thr). *RNF186* encodes a protein with a RING domain and two transmembrane domains. Importantly, the disease-coding variant is located in the RING domain, a domain with a predicted E3 ubiquitin-protein ligase activity (Fig. 1).

**Table 1:** Identification of rare variants associated with ulcerative colitis.

	Follow-up genotyping			IIBDGC Immunochip data			Combined			
Gene, mutation	alleles tested,			alleles tested,			alleles tested			
chromosome: position^a^	allele frequency^c^			allele frequency^c^						
	UC	HC	*P*	UC	HC	*P*	UC	HC	OR (95% CI)	*P*
*RNF186*, p.Ala64Thr	14580	16034	8.69E-04	NA	NA	NA	14580	16034	1.49	8.69E-04
1: 20013992	1.21%	0.69%							(1.17–1.90)	
*IL23R*, p.Gly149Arg^b^	14472	15936	0.097	14262	24346	0.197	28734	40282	0.74	0.032
1: 67421184	0.25%	0.35%		0.34%	0.44%				(0.56–0.97)	
*IL23R*, p.Val362Ile^b^	14566	16026	0.025	11182	21102	0.024	25748	37128	0.79	1.18E-03
1: 67478488	1.27%	1.52%		1.17%	1.48%				(0.68–0.91)	
*CEP72*, p.Lys314Arg	10278	10534	0.012	NA	NA	NA	10278	10534	0.17	0.012
5: 690668	0%	0.095%							(0.036–0.79)	
*CEP72*, p.Asp316Asn	14558	16034	0.043	NA	NA	NA	14558	16034	0.34	0.043
5: 690673	0.021%	0.075%							(0.12–1.00)	
*CCR6*, p.Ala369Val	13378	14454	4.52E-04	11180	21098	0.71	24560	35552	1.26	0.013
6: 167470814	0.99%	0.66%		0.84%	0.82%				(1.05–1.51)	
*LAMB1*, p.Ile1547Thr	13374	14450	0.018	11170	21090	0.159	27432	35540	1.16	0.017
7: 107357198	2.03%	1.61%		2.39%	2.10%				(1.03–1.30)	
*JAK2*, p.Arg1063His	14528	15976	0.015	NA	NA	NA	14528	15976	0.65	0.015
9: 5116343	0.34%	0.58%							(0.45–0.92)	
*CARD9*, c.IVS11+1G>C^b^	7002	7146	1.21E-06	14286	24362	1.81E-06	21290	31508	0.39	1.47E-11
9: 138379413	0.31%	0.99%		0.28%	0.71%				(0.30–0.53)	
*STAC2*, p.Lys302Arg	14580	16036	0.038	NA	NA	NA	14580	16036	1.39	0.038
17: 34624048	0.62%	0.47%							(1.02–1.90)	

a Positions from Human genome build 36.

b Previously reported variant independently identified in the current study.

c Minor allele frequencies estimates from combined case:control cohorts; actual allele frequencies can vary between cohorts.

UC, Ulcerative Colitis; HC, Healthy Controls; NA, data not available.

Independence of effect between rare variants in *IL23R* and *CARD9* and the reported common association signals in these genes has previously been shown [7], [11]. For *RNF186*, the Ala64Thr variant is mostly found on the protective haplotype background from the previously identified common variant, indicating that the reported association is not likely due to partial LD with the common variant. In addition, reciprocal conditional logistic regression analysis, using a subset of samples where both variants were genotyped (3548 UC cases and 3607 healthy controls) shows that these are independent association signals (data not shown).

Given the challenge inherent in achieving corrected significance thresholds for rare variants, even with large cohorts, we expect that some of the other variants that we identified and found to have nominal significance (0.0012<*P*<0.05) are truly associated with UC. In fact with a target set of 42 variants included in follow-up genotyping, and supposing these are independent and under the null, we would expect <1 SNP to exceed *P*<0.01 (with a probability of less than 1% to observe 3 or more associations at this level) and ∼2 SNPs to exceed *P*<0.05 by chance alone (with a probability less than 0.0001 to observe 9 or more association at this level), whereas we observe 3 SNPs with *P*<0.01 and 9 SNPs with *P*<0.05, suggesting that there are additional true positives that have not met the more stringent threshold. Indeed, within the group of SNPs that we found to have nominal significance are two non-synonymous coding variants (Gly149Arg and Val362Ile) in *IL23R* that we and others have shown to be associated with protection from IBD (Table 1) [7], [11]. In addition to these previously-validated variants in *IL23R*, we have found variants that are nominally associated with UC in the genes encoding *CEP72*, *LAMB1*, *CCR6*, *JAK2*, and *STAC2* (Table 1). Specifically, we identified two nominally associated rare variants in *CEP72* (Lys314Arg and Asp316Asn) in perfect LD with each other that appear to protect from UC (Table 1). As we also sequenced the only other gene in this locus (*TPPP*), but did not find any associated variants in it, this suggests that *CEP72* is potentially causal. Similarly, we sequenced both genes in the *LAMB1-DLD* locus on chromosome 7, with the nominally associated rare variant in *LAMB1* (Ile154Thr) suggesting a role for this gene in risk to UC, especially as the associated allele is located in its DUF287 domain and is predicted to have a damaging effect [12]. All genes within the *CCR6-FGFR1OP-RNASET2* locus were sequenced, with a single nominally-associated variant (Ala369Val) in *CCR6*, consistent with this gene's probable role in the migration and recruitment of dendritic and T cells during inflammatory and immunological responses [13]. Within the *JAK2-INSL6-LHX3* locus, we only sequenced *JAK2* given its key role in signaling from the IL12R/IL23R, a biological pathway proven to be associated with IBD, and identified a nominally associated variant (Arg1063His) within its catalytic domain. *STAC2* is within a locus with 16 other genes including *ORMDL3*, which has been suggested to be the most likely causal gene based previous genetic and functional studies in IBD and asthma [8], [14]. Although we find a nominally associated variant in *STAC2* (Lys302Arg) and none in *ORMDL3*, we have only sequenced 10 of the 17 genes within this locus (Table S4). Studies of each of these variants to determine their functional impact will be essential to prove causality.

## Discussion

Genome-wide association studies in IBD have been very successful in identifying genomic regions associated with CD, UC or both. Only infrequently have these GWA studies also directly identified the causal genes/variants, with *NOD2*, *IL23R* and *ATG16L1* being the few known examples. A recent targeted (exons and exon-intron boundaries) sequencing approach of known CD loci resulted in the identification of potentially causal variants in eight of the 36 loci examined [7]. The primary objective of the current study therefore was to use the same approach to identify likely causal variants within genes that were located in genomic regions associated with UC. While there are over 100 UC loci that have been identified and validated to date, we examined 22 UC loci that were known at the time of the initiation of this project. Of these 22 loci, the current study identified potentially causal variation in three of the loci: two protective alleles in *CARD9* and *IL23R*, and an allele increasing risk in *RNF186*.

The identification of a rare variant (Ala64Thr) in *RNF186* that shows significant association to UC strongly suggests that this is the causal gene within this locus. Importantly, the disease-coding variant is located in the RING domain, a domain with a predicted E3 ubiquitin-protein ligase activity. Ubiquitin ligases have been shown to regulate key adaptors of proinflammatory pathways [15]–[17]. We previously reported that *RNF186* expression was higher in human intestinal tissues than in immune tissues [8]. We showed by immunostaining that the RNF186 protein was expressed at the basal pole of epithelial cells and lamina propria within colonic tissues. Using GEO public microarray datasets, we pursued a systematic follow-up analysis of expression profiles of epithelial cells in response to bacterial products, PAMPs/pathogens. We found that *RNF186* gene expression was significantly up-regulated in small intestine epithelium and induced by Shigella infection in mice (*P* = 4.21×10^−8^) (Figure 1, Panel A) [18], [19]. Both invasive (INV+) and non-invasive (INV−) strains of Shigella induced significant overexpression of *RNF186* in intestinal tissues of 4-day- and 7-day-old mice infected for 2 or 4 hours. To further identify putative transcriptional regulators of *RNF186* expression, we employed a text-mining and network-generating analysis of human protein-protein, protein-DNA, protein-RNA and protein-compound interactions. Specifically, from our analyses we hypothesize that *RNF186* is transcriptionally regulated in a two-step process by the transcription factor Hepatocyte Nuclear Factor 4, alpha (HNF4A) (Figure 1, Panels B,C). Several studies have shown that HNF4A binds to the promoter region and up-regulates the expression of yet another transcription factor *HNF1A* [20]–[22]. Knockdown of *HNF4A* has been shown to down-regulate *HNF1A* gene expression [23], [24]. HNF1A, in turn, regulates *RNF186* and this interaction has been confirmed by chromatin immunoprecipitation and chip-on-chip assay [25]–[27]. Our own analysis of transcriptional profiles of HNF4A-Null colons recovered from HNF4A^loxP/loxP^ *Foxa3Cre* and HNF4A^loxP/−^ *Foxa3Cre* mice uncovered a significant up-regulation of *RNF186* transcript [28]. Expression profiling of human tissues also supports this hypothesis, as *HNF4A* and *RNF186* are clearly co-expressed in the small intestine and the colon (Figure S1). This putative interaction is particularly relevant given that *HNF4A* has previously been shown to be associated, with genome-wide significance, with risk to developing UC [9]. Our analysis now indicates a direct genetic interaction between two IBD susceptibility genes namely, *HNF4A* and *RNF186*. While a singular loss-of-function mutation in *HNF4A* has already been shown to be associated with susceptibility to abnormal intestinal permeability, inflammation and oxidative stress, we speculate that a dual loss-of-function with additional mutation in *RNF186* would further exacerbate one's susceptibility to develop chronic inflammation in the gut [29], [30].

In addition to the variants in *IL23R*, *CARD9*, and *RNF186*, we also identified variants of interest in an additional five loci (specifically within the *CEP72*, *LAMB1*, *CCR6*, *JAK2*, and *STAC2* genes). While these latter six still require confirmation, we estimate that many will validate given that we observed an excess of nominally-associated variants. Examining the data from the current study along with the data derived from prior association and sequencing studies suggests that at a minimum, there currently is strong evidence of association to causal variation in IBD (i.e. missense, nonsense or splice junction variants) in the *NOD2*, *ATG16L1*, *IL23R*, *MST1*, *CARD9*, *IL18RAP* and *RNF186* genes, and at least suggestive evidence for causal variation in the *CUL2*, *C1orf106*, *PTPN22*, *MUC19*, *CEP72*, *LAMB1*, *CCR6*, *JAK2*, and *STAC2* genes (Current study and references [4], [5], [7], [11], [31]). While only a small fraction of the recently identified 163 IBD loci have been sequenced (36 CD, 22 UC for total of 42 independent loci) in IBD patients and controls, this would suggest that from ∼10% (15 of 163 total loci) to ∼35% (15 of 42 loci sequenced) of IBD loci have causal variation affecting the protein-coding or splice junctions. There are an additional 5 loci (*ITLN1*, *GSDMB*, *YDGL*, *SLC22A4*, and *FCGR2A*) for which there are non-synonymous coding or splice variants present in public databases (dbSNP, 1KG) that are correlated with the index SNP identified in the GWA studies that have yet been tested directly, thus potentially increasing the estimated number of IBD loci with causal variation within the coding and splice regions [3], [32].

Furthermore, it should be noted that with the exception of a small number of variants with significant effect (e.g. R702W, G908R, fs107insC in NOD2; R381Q in IL23R; IVS11+1G>C in CARD9; V527L in IL18RAP – all of which had 0.5>OR>2) most of the rare variants identified by targeted sequencing of loci from GWAS regions have relatively modest effect sizes that are comparable to those observed for the common variants identified by GWA studies. Consequently, very large sample sizes are required to detect statistically significant association. In the current study, for the majority (93%) of variants with an observed minor allele frequency greater than 0.3%, we had more than 80% power to detect significant association if the OR is 2 or greater with the number of samples typed (up to ∼14,000 cases and ∼20,000 controls) (see Table S5). Moreover, should this observation not be limited to risk loci identified by GWA studies, this has implications with respect to future efforts for discovering risk loci. Specifically, if the occurrence of rare variants with large effects sizes is relatively infrequent, then this may favor the current paradigm of locus discovery by GWA followed by targeted sequencing rather than whole-exome or whole-genome sequencing for locus discovery as this would require even larger sample sizes. Alternatively, given the ever- growing size of public databases of common and rare variants, targeted genotyping of known variants within risk loci identified by GWA may prove to be an efficient approach. For example, all but two of the 22 candidate causal variants identified in the current study or that of Rivas and colleagues are now found in the Exome Sequencing Project database.

Regardless of the study design, these results suggest that a significant proportion of IBD loci contain causal variants within exons or exon-intron boundaries. While these rare/infrequent variants may not account for what has been termed “the missing heritability” of common traits, discovering these variants certainly can provide focus for follow-up functional studies. For example, the current sequencing and follow-up genotyping of the chromosome 1p36 locus, which was first identified in a GWA study of UC, identified significant association to the Ala64Thr variant within *RNF186*. While further studies will be required, the initial bioinformatics and experimental studies described above suggest that this ring finger protein with an ubiquitin-ligase domain may have an important role in the response to microbes/microbial products. Going forward, systematic evaluation of genes within risk loci via expression-driven functional studies in cellular models (i.e. knock-down or over expression) with sensitive high throughput/high content readouts may very well be a complementary approach given that at least a third of IBD risk loci appear to act via gene expression [3].

## Materials and Methods

### DNA preparation and pooling

We selected 200 ulcerative colitis patients and 150 healthy control of French-Canadian descent from the NIDDK IBD Genetics Consortium repository samples. The NIDDK IBDGC samples were collected under rigorous clinical phenotyping and control matching for the purpose of genetic studies [33]. Genomic DNA concentrations were measured by Quant-iT PicoGreen dsDNA reagent (Invitrogen) and detected on the Biotek Synergy 2 plate reader. All DNAs were normalized with at least two round of dilution and quantification down to a concentration of 10 ng/µl as described previously [7]. Equimolar amounts of samples were pooled together in batches of 50 cases and 50 controls for a total of 7 pooled groups.

### Target selection and design

Target exonic sequences were selected based on the coding exons of 55 genes in 14 UC-associated regions and 7 regions identified in CD with nominal replication in our recent UC GWAS study, as well *ECM1* identified from recent candidate-gene study in UC [6], [8]–[10], [34]. Specifically, amplicons were designed from genome build Hg18 using a web-base automated pipeline (Optimus primer: Website (http://op.pgx.ca)) that uses the Primer 3 design software and user defined parameters [35]. Design parameters included amplicon sizes between 400 and 600 base pairs, as well as the inclusion of *Not1* tails for subsequent concatenation and shearing steps in library construction. PCR amplification reactions contained 40 ng of pooled genomic DNA, 1× HotStar buffer, 0.8 mM dNTPs, 2 mM MgCl2, 0.4 units of HotStar Enzyme (Qiagen), and 0.25 µM forward and reverse primers in a 10-µl reaction volume. PCR cycling parameters were as follows: one cycle of 95°C for 5 min; 30 or 35 cycles of 94°C for 30 s, 60°C for 30 s, and 72°C for 1 min; followed by one cycle of 72°C for 5 min. Each DNA pools were amplified for 508 PCR reactions; amplification products were then dosed by Quant-iT PicoGreen dsDNA reagent (Invitrogen) quantification and amplification specificity was validated by agarose gel electrophoresis. In total, 472 PCR amplicons (93% amplification success rate, capturing 283 Kb including 117 Kb of target exonic sequences) (Table S6) for each DNA pool were combined in equimolar amounts to obtain equal representation of all target in library construction.

### Sequencing and variant discovery

The combined PCR products from each pooled DNA group were concatenated using the *NotI* adapters and sheared into fragments as previously described [36]. Libraries were constructed according to Illumina single-end library protocol, with 150–200 bp gel size selection and PCR enrichment using 10 cycles of PCR, and then single-end sequenced with 36 cycles on an Illumina Genome Analyzer II. Each sample pool was sequenced using a single lane of Illumina GAII analyzer flowcell; 36-base pair reads were aligned to the genome using MAQ algorithm [37] and base qualities were recalibrated using GATK (Genome Analysis ToolKit) [38]. Finally, variant discovery was performed using the previously described Syzygy software, designed to analyze sequencing data from pooled DNA sequencing [7].

### Genotyping, validation and follow-up genotyping

We randomly selected 237 high quality variants for validation in our 350 discovery DNAs samples using Sequenom MassARRAY iPlex200 chemistry. Genotyping assay designs were obtained from the Assay Designer v.3.1 software, and genotyping oligonucleotides were synthesized at Integrated DNA Technologies. The correlation coefficient between observed minor allele frequencies and frequencies estimated from Syzygy for validated variants was calculated in order to evaluate the overall quality of our dataset (Figure S2). Eighty-four high quality non-synonymous coding variants (missense, nonsense and splicing variants (within 2 bp of a splice site)) remained after the exclusion of singletons from our sequencing results, variants that did not validate and variants within the MHC region. We then evaluated these variants in an independent cohort of North-American individual of European descent from the NIDDK IBD genetics consortium (754 cases and 1008 controls); only variants detected in this independent cohort were kept for follow-up genotyping. Following assay design, 42 SNPs were genotyped using Sequenom MassARRAY iPlex200 chemistry in 6 independent follow-up case-control cohorts (7292 cases and 8018 controls) (Table S3). Because of design constraints and assay failures, not all markers were examined in all follow-up sample sets. For a subset of these variants, further genotyping data was obtained from the International IBD Genetics Consortium Immunochip data (7143 UC, 12186 controls)

### Cohort descriptions

For all cohorts, UC was diagnosed according to accepted clinical, endoscopic, radiological and histological findings.

Genotyping of the NIDDK IBDGC cohort, as well as the Italian and Dutch cohorts was performed at the Laboratory for Genetics and Genomic Medicine of Inflammation (www.inflammgen.org) of the Université de Montréal.

NIDDK IBD Genetics Consortium (IBDGC) samples were recruited by the centers included in the NIDDK IBDGC: Cedars Sinai, Johns Hopkins University, University of Chicago and Yale, University of Montreal, University of Pittsburgh and University of Toronto. Additional samples were obtained from the Queensland Institute for Medical Research, Emory University and the University of Utah. Medical history was collected with standardized NIDDK IBDGC phenotype forms. Healthy controls are defined as those with no personal or family history of IBD.

The Italian samples were collected at the S. Giovanni Rotondo “CSS” (SGRC) Hospital in Italy.

The Dutch cohort is composed of ulcerative colitis cases recruited through the Inflammatory Bowel Disease unit of the University Medical Center Groningen (Groningen), the Academic Medical Center (Amsterdam), the Leiden University Medical Center (Leiden) and the Radboud University Medical Center (Nijmegen), and of healthy controls (n = 804) of self-declared European ancestry from volunteers at the University Medical Center (Utrecht).

Genotyping of the German cohort was performed at the Institute for Clinical Molecular Biology

Christian-Albrechts-University in Kiel. German patients were recruited either at the Department of General Internal Medicine of the Christian-Albrechts-University Kiel, the Charité University Hospital Berlin, through local outpatient services, or nationwide with the support of the German Crohn and Colitis Foundation. German healthy control individuals were obtained from the popgen biobank.

Genotyping of Swedish UC cases and controls was performed at Karolinska Institutet's Mutational Analysis core facility (MAF). Swedish ulcerative colitis patients and controls were recruited at the Karolinska University Hospital, Stockholm, and at the Örebro University Hospital, Örebro, Sweden.

Genotyping of the Belgian cohort was performed at the Genomics Core Facility at UZ Leuven, using a MassARRAY iPLEX (Sequenom). Belgian patients were all recruited at the IBD unit of the University Hospital Leuven, Belgium; control samples are all unrelated, and without family history of IBD or other immune related disorders.

### Ethics statement

All patients and control subjects provided informed consent. Recruitment protocols and consent forms were approved by Institutional Review Boards at each participating institutions. All DNA samples and data in this study were denominalized.

### Association analysis

Association analysis of follow-up genotyping data was performed using the previously described mega-analysis of rare variants (MARV) approach [7]. Briefly, this method evaluates significance of association from stratified sample, using within sub-cohort permutation of individual phenotypes to provide the test statistic. This approach is robust to population stratification and to deviation from Hardy-Weinburg equilibrium.

### Network analyses

We downloaded and analyzed several Gene Expression Omnibus (GEO) public microarray datasets including: (a) Expression data from newborn mice infected with Shigella flexneri; GSE9785 (b) Transcription profiles of colon biopsies from UC patients and healthy controls; GSE11223 (c) Steady-state gene expression data of Tuberculosis infected human primary dendritic cells; GSE34151 (d) PBMC transcriptional profiles in healthy subjects, patients with Crohn's Disease, and patients with Ulcerative Colitis; GSE3365, (e) Transcription profiles of colon biopsies from Crohn's patients and healthy controls; GSE20881, (f) Transcription profile of mouse small intestine epithelium vs. mesenchyme; GSE6383, (g) Gene expression in *HNF4* null mouse colons compared to control colons; GSE3116, and (h) Microarray profiles of mouse epithelial colon harboring conditional knock out of *HFN4A*; GSE11759. Each of these datasets was normalized using quantile normalization routine in MATLAB. Genes were tested for significant differences between pairs of control and stimulated/treated samples within each experiment. After selecting genes with nominal *P*<0.05, estimated using an unpaired T-test, expression of *RNF186* was evaluated whether it passed the significance threshold or not. The results of processing all these datasets are shown in Table S7 and Figures S3, S4, S5, S6, S7, S8, S9, S10, S11, S12, S13, S14. For transcriptional network analysis, we used Metacore's suite of network building algorithms to expand the sub-network around RNF186. The algorithm searches through a manually curated knowledgebase of molecular interaction to identify bidirectional connectivity with genes, proteins and small molecules. The search was constrained to expand the overall network size up to 50 components. Given that the bioinformatic analyses suggested that HNF4A controlled the expression of *RNF186*, we directly tested for their co-expression in a panel of RNAs from a variety of human tissues. Specifically, expression levels of *RNF186* and *HNF4A* were evaluated using a custom expression array from Agilent, which was designed to include an independent probe for each exon of the genes tested (Figure S1). Briefly, total RNA from bone marrow, heart, skeletal muscle, uterus, liver, fetal liver, spleen, thymus, thyroid, prostate, brain, lung, small intestine and colon were purchased from Clontech Laboratories. A reference RNA sample was also included that consisted of an equal mix from 10 different human tissues (adrenal gland, cerebellum, whole brain, heart, liver, prostate, spleen, thymus, colon, bone marrow). With the exception of the small intestine (RIN = 7.6), all RNAs had a RNA Integrity Value (RIN) value ≥8 (range 8.0–9.3) as measured by Agilent 2100 Bioanalyzer using the RNA Nano 6000 kit (Agilent Technologies). Labeled cRNA was then synthesized from 50 ng of each RNA sample using the Low Input Quick Amp WT labeling kit (Agilent Technologies) according to the manufacturer's protocol. Quantity and quality of labeled cRNA samples were assessed by NanoDrop UV-VIS Spectrophotometer. Sample hybridization was performed according to the manufacturer's standard protocol and microarrays were scanned using the Sure Scan Microarray Scanner (Agilent technologies). An expression value was obtained for each gene in each replicate by calculating the geometric mean of all probes within the gene, followed by a median normalization across all genes on the array. A geometric mean and geometric standard deviation was calculated from at least 3 independent measurements for each tissue.

## Supporting Information

Figure S1 *RNF186* and *HNF4A* are co-expressed in human intestinal tissues. Expression levels of (A) *RNF186* and (B) *HNF4A* were evaluated in a panel of human tissues (bone marrow (Bone M.), heart, skeletal muscle (Sk.Muscle), uterus, liver, fetal liver (F.Liver), spleen, thymus, thyroid, prostate, brain, lung, small intestine (Small I.) and colon) and shown to be co-expressed in small intestine and colon, but show differential expression in liver. Intensity values for each tissue represent the geometric mean with geometric standard deviation of 3 independent measurements; each measurement represents the geometric mean of all probes (one per exon) for each gene followed by a median normalization across all genes on the array. The dotted line indicates the threshold level for detection of basal expression. The reference sample (Ref.) is composed of a mixture RNAs derived from 10 different human tissues.
(TIFF)

Figure S2 Correlation between minor allele frequencies estimated from sequence and genotype data. Minor allele frequency correlation between Syzygy estimates and genotyped data in discovery samples for 179 non-monomorphic variants from the 237 randomly selected set of high quality variant. (A) Whole range of minor allele frequencies shown. (B) Infrequent allele frequencies only (minor allele frequency ≤0.05). In this experiment, correlation gets lower as minor allele frequency threshold increase (R^2^ = 0.88, 0.75, 0.59, 0.57 and 0.19 for MAF≥0, 0.05, 0.10, 0.20 and 0.30, respectively). This reflects the increase in absolute error for variants with greater MAF (funnel shaped plot), and is consistent with the higher validation rate for low-frequency variants (Table S2).
(TIFF)

Figure S3 Comparative gene expression profiling in PBMC from healthy subjects and patients with ulcerative colitis (UC). (A) Table of gene expression fold change statistics from comparison of PBMC transcriptional profiles in healthy subjects and patients with ulcerative colitis (UC) (GSE3365). Only top 10 genes and *RNF186* are shown. The rank column refers to the rank of the gene for signal to noise ratio in the specific study (1061 significant genes ranked). (B) Plot of *RNF186* gene expression in samples from normal individuals and patients with UC. The squares and crosses represent median and mean respectively.
(TIFF)

Figure S4 Comparative gene expression profiling in PBMC from healthy subjects and Crohn's disease patients. (A) Table of gene expression fold change statistics from comparison of PBMC transcriptional profiles in healthy subjects and Crohn's disease patients (GSE3365). Only top 10 genes and *RNF186* are shown. The rank column refers to the rank of the gene for signal to noise ratio in the specific study (1844 significant genes ranked). (B) Plot of *RNF186* gene expression in samples from normal individuals and Crohn's disease patients. The squares and crosses represent median and mean respectively.
(TIFF)

Figure S5 Comparative gene expression profiling in colon epithelial biopsies from ulcerative colitis patients and healthy control donors. (A) Table of gene expression fold change statistics from transcriptional profiling of colon epithelial biopsies from ulcerative colitis patients and healthy control donors (GSE11223). Only top 10 genes and *RNF186* are shown. The rank column refers to the rank of the gene for signal to noise ratio in the specific study (1214 significant genes ranked). (B) Plot of *RNF186* gene expression in samples from Non-inflamed control colon and inflamed colon. The squares and crosses represent median and mean respectively.
(TIFF)

Figure S6 Comparative gene expression profiling of endoscopic biopsies taken at ileocolonoscopy from ascending colon of Crohn's disease patients and healthy control donors. (A) Table of gene expression fold change statistics from transcriptional profiling of endoscopic biopsies taken at ileocolonoscopy from ascending colon of Crohn's disease patients and healthy control donors (GSE20881). Only top 10 genes and *RNF186* are shown. The rank column refers to the rank of the gene for signal to noise ratio in the specific study (2510 significant genes ranked). (B) Plot of *RNF186* gene expression in samples from ascending colon biopsies of normal subjects and Crohn's disease patients. The squares and crosses represent median and mean respectively.
(TIFF)

Figure S7 Comparative gene expression profiling of endoscopic biopsies taken at ileocolonoscopy from descending colon of Crohn's disease patients and healthy control donors. (A) Table of gene expression fold change statistics from transcriptional profiling of endoscopic biopsies taken at ileocolonoscopy from descending colon of Crohn's disease patients and healthy control donors (GSE20881). Only top 10 genes and *RNF186* are shown. The rank column refers to the rank of the gene for signal to noise ratio in the specific study (579 significant genes ranked). (B) Plot of *RNF186* gene expression in samples from descending colon biopsies of normal subjects and Crohn's disease patients. The squares and crosses represent median and mean respectively.
(TIFF)

Figure S8 Comparative gene expression profiling of endoscopic biopsies taken at ileocolonoscopy from sigmoid colon of Crohn's disease patients and healthy control donors. (A) Table of gene expression fold change statistics from transcriptional profiling of endoscopic biopsies taken at ileocolonoscopy from sigmoid colon of Crohn's disease patients and healthy control donors (GSE20881). Only top 10 genes and *RNF186* are shown. The rank column refers to the rank of the gene for signal to noise ratio in the specific study (613 significant genes ranked). (B) Plot of *RNF186* gene expression in samples from sigmoid colon biopsies of normal subjects and Crohn's disease patients. The squares and crosses represent median and mean respectively.
(TIFF)

Figure S9 Comparative gene expression profiling of endoscopic biopsies taken at ileocolonoscopy from terminal ileum of Crohn's disease patients and healthy control donors. (A) Table of gene expression fold change statistics from transcriptional profiling of endoscopic biopsies taken at ileocolonoscopy from terminal ileum of Crohn's disease patients and healthy control donors (GSE20881). Only top 10 genes and *RNF186* are shown. The rank column refers to the rank of the gene for signal to noise ratio in the specific study (2608 significant genes ranked). (B) Plot of *RNF186* gene expression in samples from terminal ileum biopsies of normal subjects and Crohn's disease patients. The squares and crosses represent median and mean respectively.
(TIFF)

Figure S10 Comparative gene expression profiling of murine small intestinal epithelium and mesenchyme. A) Table of gene expression fold change statistics from transcriptional profiling of murine small intestinal epithelium and mesenchyme (GSE6383). Only top 10 genes and *RNF186* are shown. The rank column refers to the rank of the gene for signal to noise ratio in the specific study (7239 significant genes ranked). (B) Plot of *RNF186* gene expression in samples from murine small intestinal epithelium and mesenchyme. The squares and crosses represent median and mean respectively.
(TIFF)

Figure S11 Comparative gene expression profiling of intestinal tissues of 4-day- or 7-day-old mice infected or not with invasive or non-invasive shigella. (A) Table of gene expression fold change statistics from transcriptional profiling of intestinal tissues of 4-day- or 7-day-old mice infected or not with invasive (INV+) or non-invasive (INV−) (GSE9785). Only top 10 genes and *RNF186* are shown. The rank column refers to the rank of the gene for signal to noise ratio in the specific study (2258 significant genes ranked). (B) Plot of *RNF186* gene expression in mice intestinal tissue infected with shigella and control samples. The squares and crosses represent median and mean respectively.
(TIFF)

Figure S12 Comparative gene expression profiling in primary dendritic cells from 65 individuals, before and after infection with MTB. (A) Table of gene expression fold change statistics from transcriptional profiles in primary dendritic cells from 65 individuals, before and after infection with MTB (GSE34151). Only top 10 genes and *RNF186* are shown. The rank column refers to the rank of the gene for signal to noise ratio in the specific study (4279 significant genes ranked). (B) Plot of *RNF186* gene expression in primary dendritic cells from 65 individuals, before and after infection with MTB. The squares and crosses represent median and mean respectively.
(TIFF)

Figure S13 Comparative gene expression profiling in HNF4a mutant and control murine colons. (A) Table of gene expression fold change statistics from comparison of transcriptional profiles in HNF4a mutant and control murine colons (GSE3116). Only top 10 genes and *RNF186* are shown. The rank column refers to the rank of the gene for signal to noise ratio in the specific study (895 significant genes ranked). (B) Plot of *RNF186* gene expression in HNF4a mutant and control murine colons. The squares and crosses represent median and mean respectively.
(TIFF)

Figure S14 Comparative gene expression profiling in mouse epithelial colons with or without conditional knock out of HNF4. (A) Table of gene expression fold change statistics from comparison of transcriptional profiles in mouse epithelial colons with or without conditional knock out of HNF4 (GSE11759). Only top 10 genes and *RNF186* are shown. The rank column refers to the rank of the gene for signal to noise ratio in the specific study (2177 significant genes ranked). (B) Plot of *RNF186* gene expression in HNF4a conditional knock out and control murine colons. The squares and crosses represent median and mean respectively.
(TIFF)

Table S1 Summary of Pooled Sequencing Experiment.
(XLSX)

Table S2 Validation of high quality variants identified by Syzygy.
(XLSX)

Table S3 Cohort descriptions.
(XLSX)

Table S4 Details of sequencing and follow-up genotyping results, as well as association analyses for each SNP tested in this study.
(XLSX)

Table S5 Power calculations for each SNP tested with observed minor allele frequency greater than 0.0001.
(XLSX)

Table S6 Sequencing coverage per gene.
(XLSX)

Table S7 Table of datasets available in public domain that were processed and analyzed for *RNF186* expression.
(XLSX)
