# Supplementary figures and images for: Deep Resequencing of GWAS Loci Identifies Rare Variants in CARD9, IL23R and RNF186 That Are Associated with Ulcerative Colitis
Source: PLoS Genet. 2013 Sep 12;9(9):e1003723. doi: 10.1371/journal.pgen.1003723 (PMC3772057; doi:10.1371/journal.pgen.1003723)

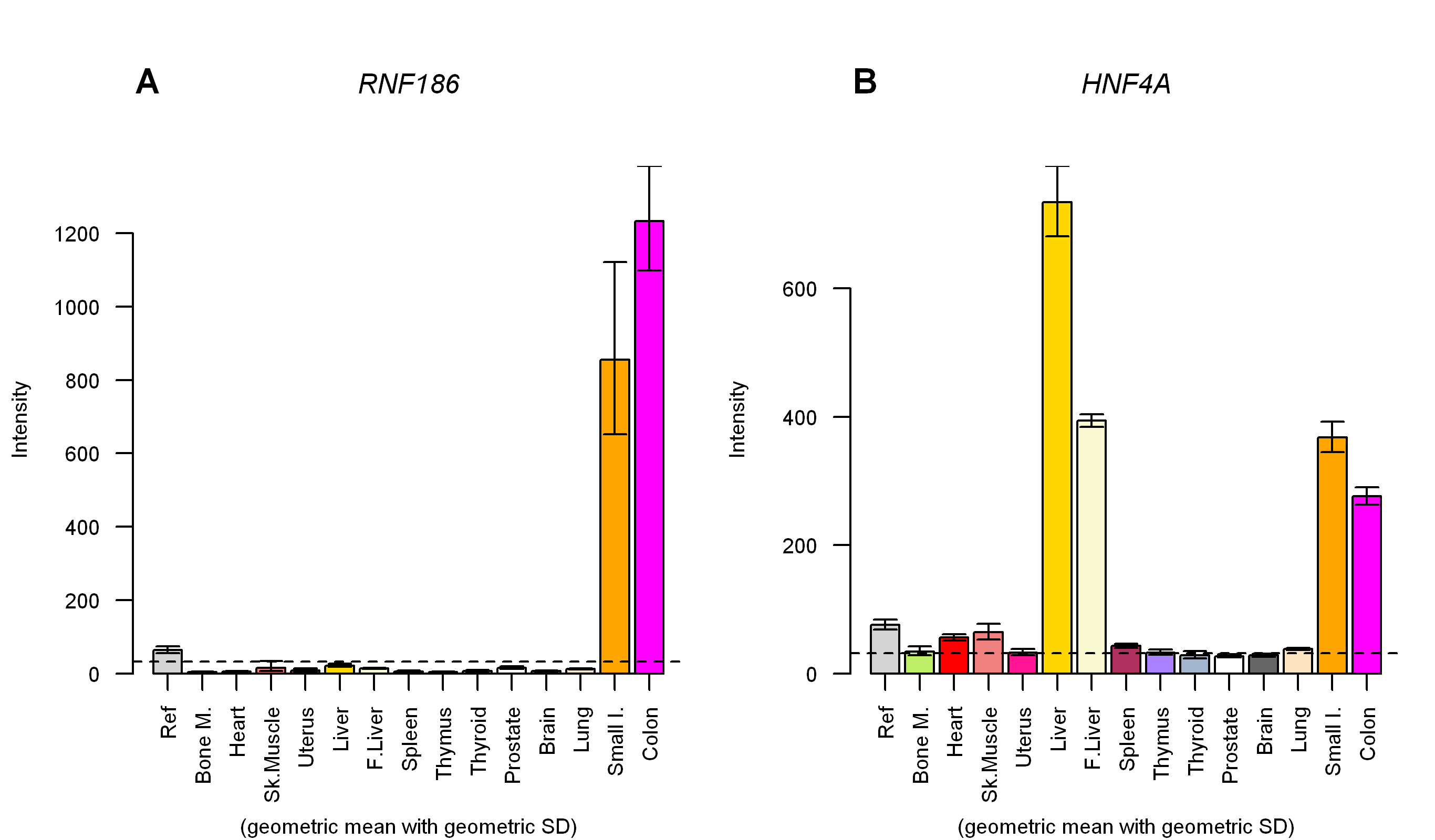

Supplement: Figure S1 — RNF186 and HNF4A are co-expressed in human intestinal tissues. Expression levels of (A) RNF186 and (B) HNF4A were evaluated in a panel of human tissues (bone marrow (Bone M.), heart, skeletal muscle (Sk.Muscle), uterus, liver, fetal liver (F.Liver), spleen, thymus, thyroid, prostate, brain, lung, small intestine (Small I.) and colon) and shown to be co-expressed in small intestine and colon, but show differential expression in liver. Intensity values for each tissue represent the geometric mean with geometric standard deviation of 3 independent measurements; each measurement represents the geometric mean of all probes (one per exon) for each gene followed by a median normalization across all genes on the array. The dotted line indicates the threshold level for detection of basal expression. The reference sample (Ref.) is composed of a mixture RNAs derived from 10 different human tissues. (TIFF) [file pgen.1003723.s001.tiff]

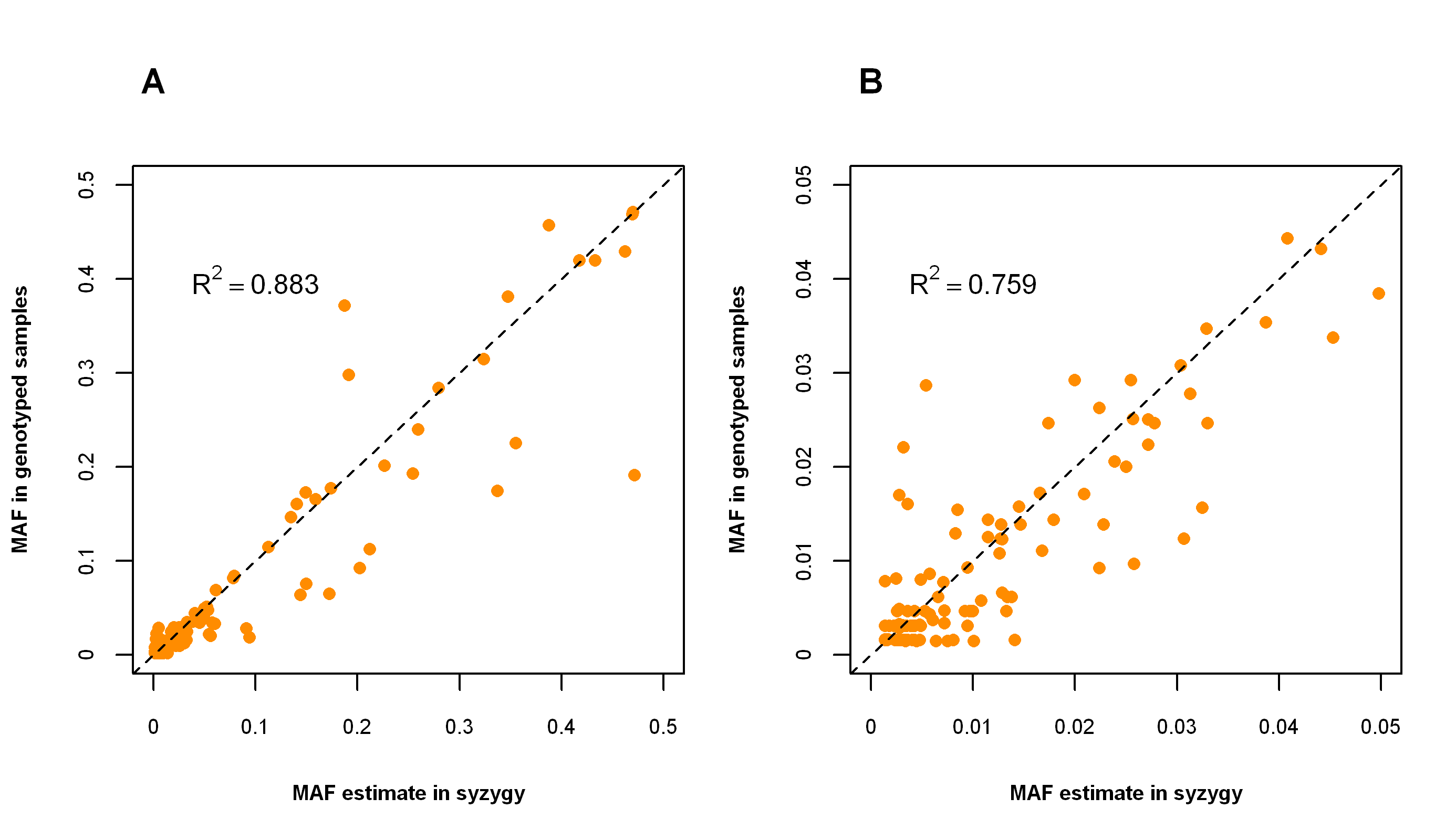

Supplement: Figure S2 — Correlation between minor allele frequencies estimated from sequence and genotype data. Minor allele frequency correlation between Syzygy estimates and genotyped data in discovery samples for 179 non-monomorphic variants from the 237 randomly selected set of high quality variant. (A) Whole range of minor allele frequencies shown. (B) Infrequent allele frequencies only (minor allele frequency ≤0.05). In this experiment, correlation gets lower as minor allele frequency threshold increase (R2 = 0.88, 0.75, 0.59, 0.57 and 0.19 for MAF≥0, 0.05, 0.10, 0.20 and 0.30, respectively). This reflects the increase in absolute error for variants with greater MAF (funnel shaped plot), and is consistent with the higher validation rate for low-frequency variants (Table S2). (TIFF) [file pgen.1003723.s002.tiff]

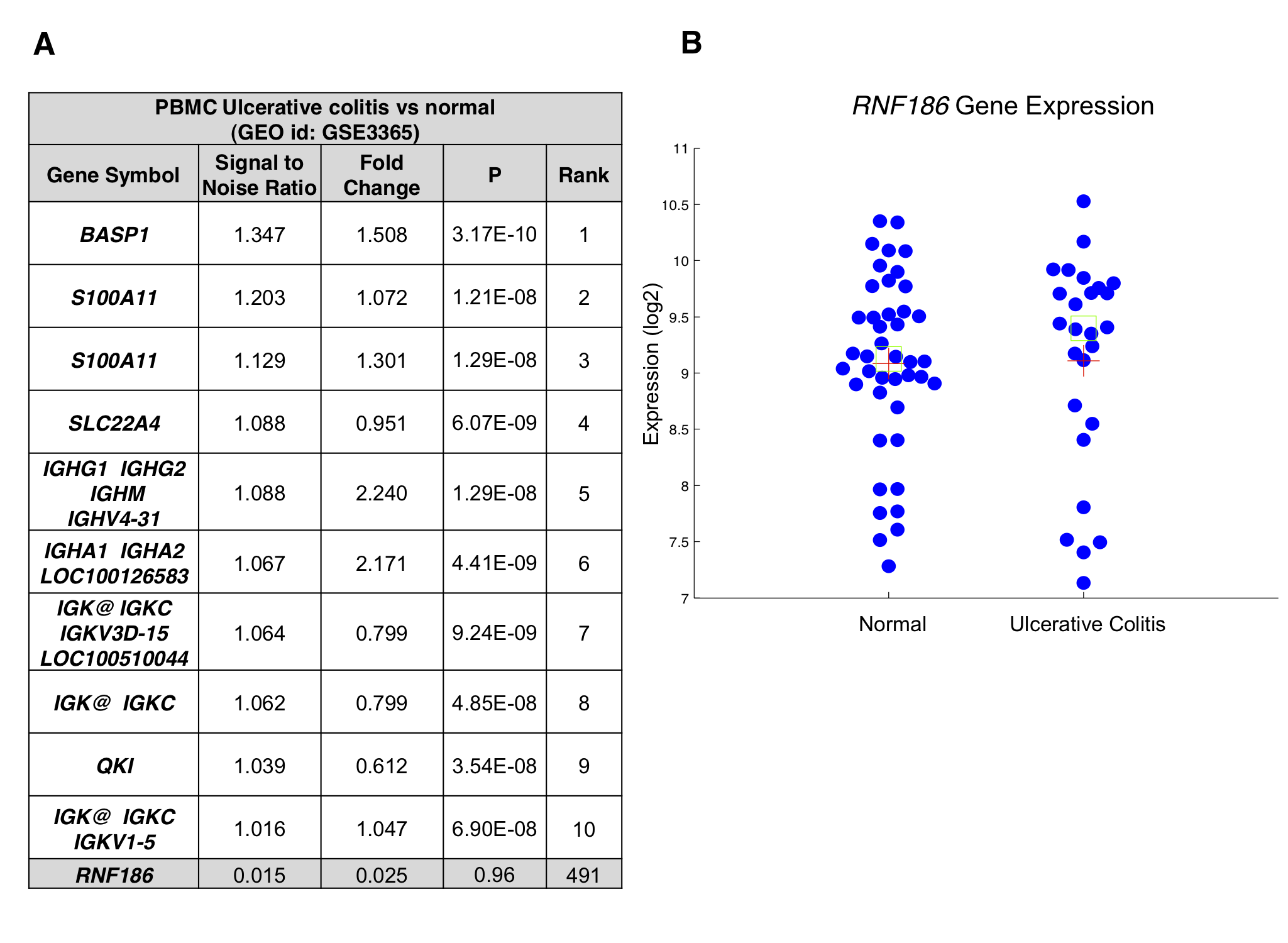

Supplement: Figure S3 — Comparative gene expression profiling in PBMC from healthy subjects and patients with ulcerative colitis (UC). (A) Table of gene expression fold change statistics from comparison of PBMC transcriptional profiles in healthy subjects and patients with ulcerative colitis (UC) (GSE3365). Only top 10 genes and RNF186 are shown. The rank column refers to the rank of the gene for signal to noise ratio in the specific study (1061 significant genes ranked). (B) Plot of RNF186 gene expression in samples from normal individuals and patients with UC. The squares and crosses represent median and mean respectively. (TIFF) [file pgen.1003723.s003.tiff]

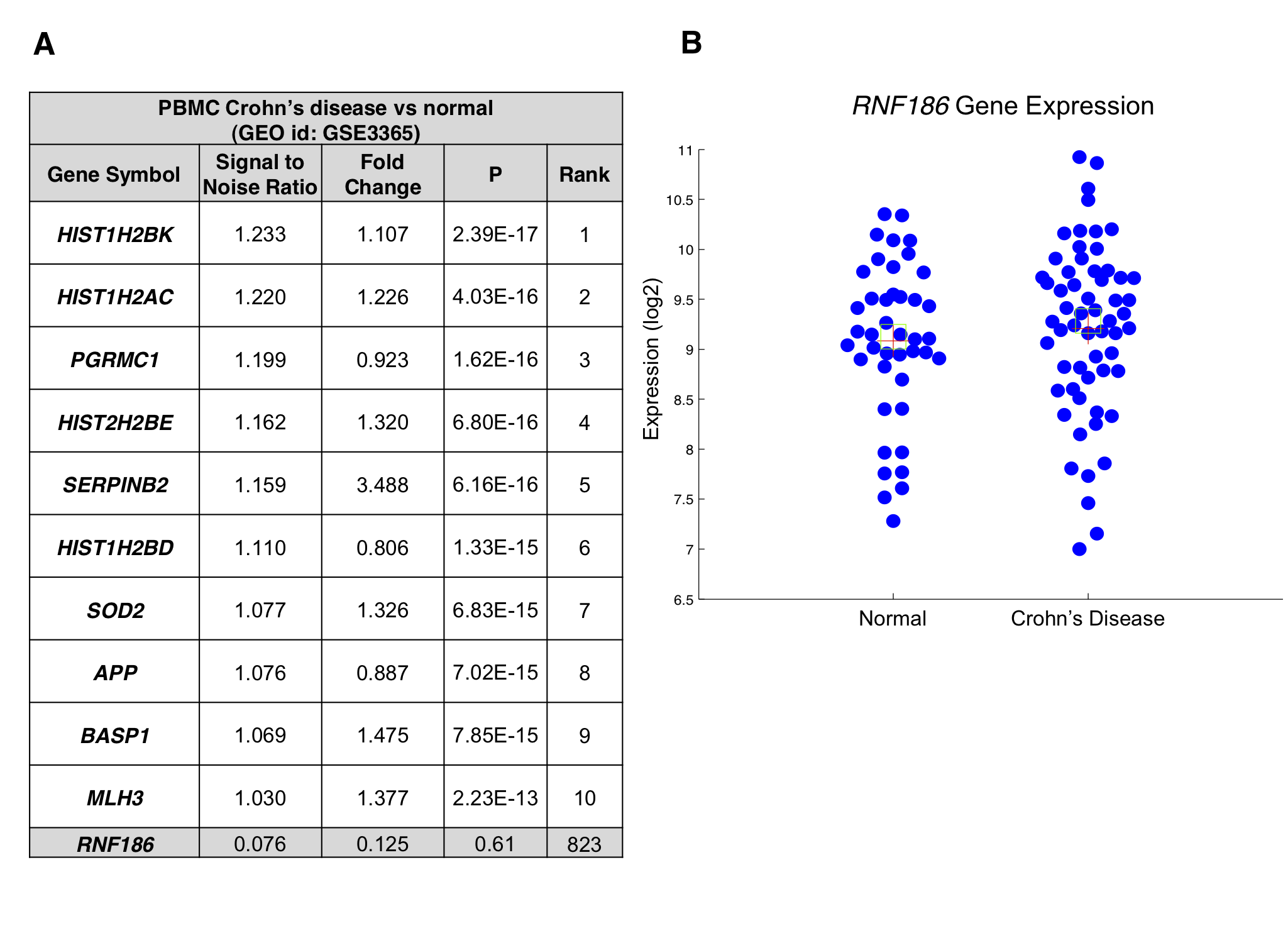

Supplement: Figure S4 — Comparative gene expression profiling in PBMC from healthy subjects and Crohn's disease patients. (A) Table of gene expression fold change statistics from comparison of PBMC transcriptional profiles in healthy subjects and Crohn's disease patients (GSE3365). Only top 10 genes and RNF186 are shown. The rank column refers to the rank of the gene for signal to noise ratio in the specific study (1844 significant genes ranked). (B) Plot of RNF186 gene expression in samples from normal individuals and Crohn's disease patients. The squares and crosses represent median and mean respectively. (TIFF) [file pgen.1003723.s004.tiff]

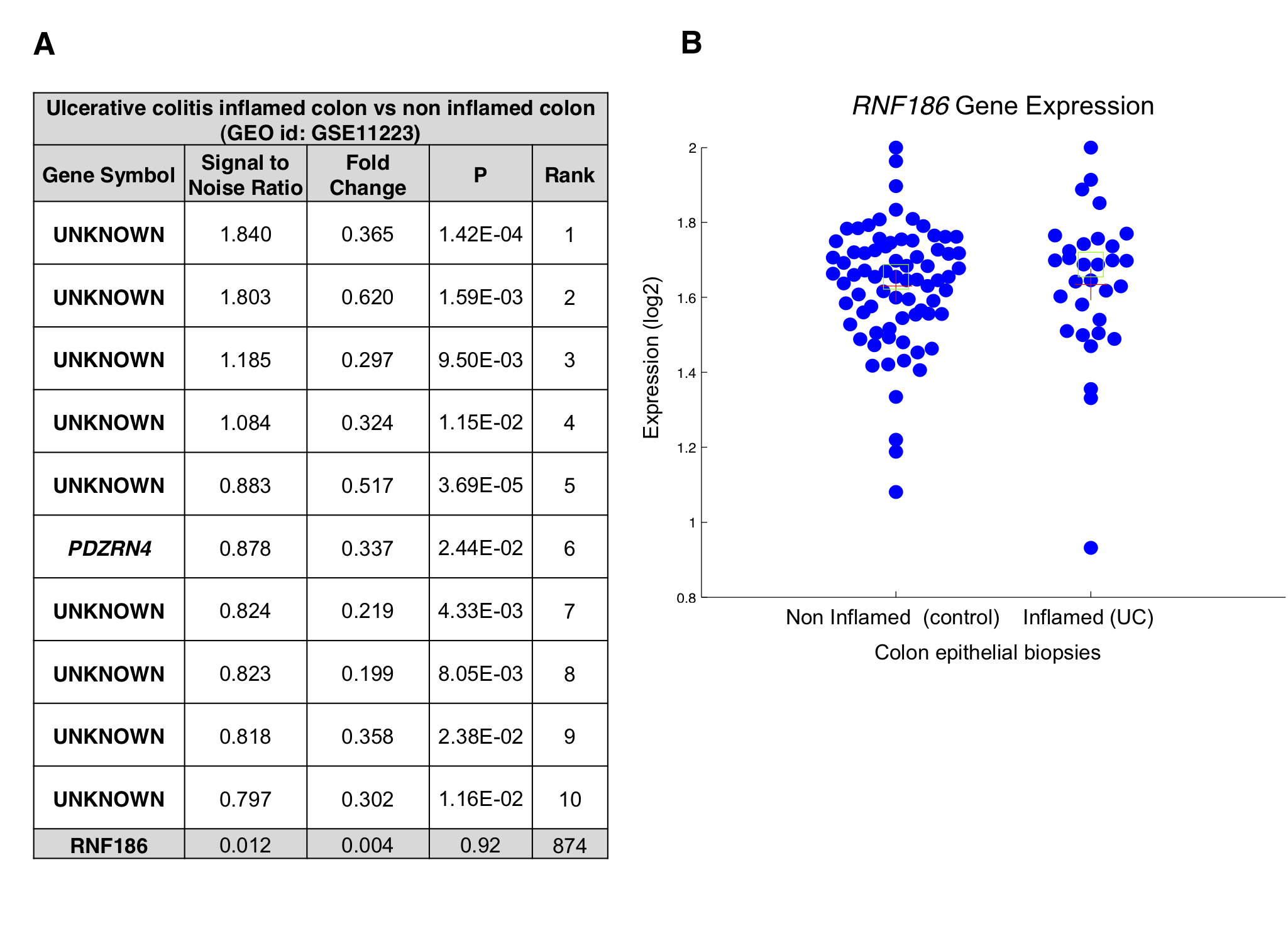

Supplement: Figure S5 — Comparative gene expression profiling in colon epithelial biopsies from ulcerative colitis patients and healthy control donors. (A) Table of gene expression fold change statistics from transcriptional profiling of colon epithelial biopsies from ulcerative colitis patients and healthy control donors (GSE11223). Only top 10 genes and RNF186 are shown. The rank column refers to the rank of the gene for signal to noise ratio in the specific study (1214 significant genes ranked). (B) Plot of RNF186 gene expression in samples from Non-inflamed control colon and inflamed colon. The squares and crosses represent median and mean respectively. (TIFF) [file pgen.1003723.s005.tiff]

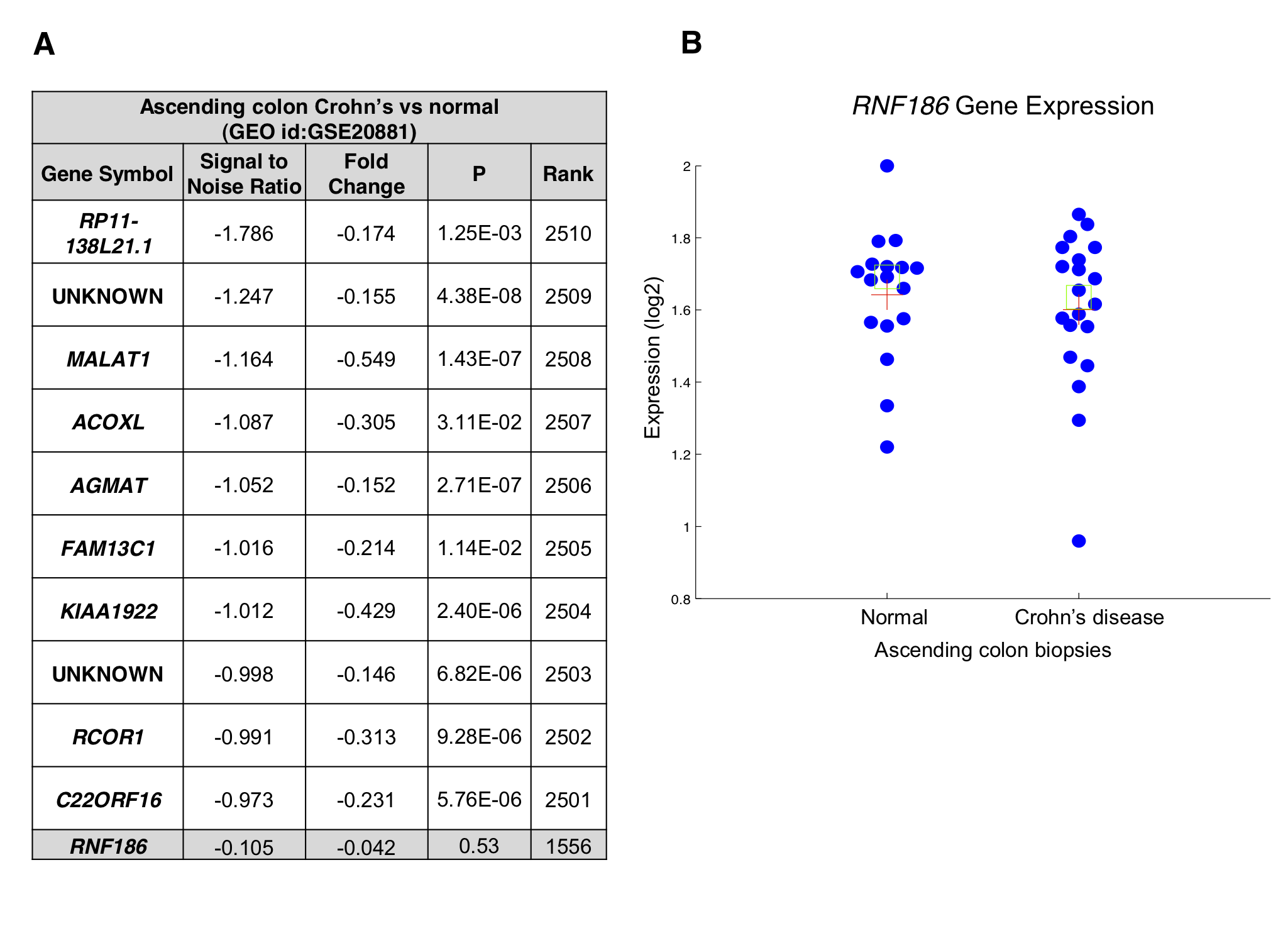

Supplement: Figure S6 — Comparative gene expression profiling of endoscopic biopsies taken at ileocolonoscopy from ascending colon of Crohn's disease patients and healthy control donors. (A) Table of gene expression fold change statistics from transcriptional profiling of endoscopic biopsies taken at ileocolonoscopy from ascending colon of Crohn's disease patients and healthy control donors (GSE20881). Only top 10 genes and RNF186 are shown. The rank column refers to the rank of the gene for signal to noise ratio in the specific study (2510 significant genes ranked). (B) Plot of RNF186 gene expression in samples from ascending colon biopsies of normal subjects and Crohn's disease patients. The squares and crosses represent median and mean respectively. (TIFF) [file pgen.1003723.s006.tiff]

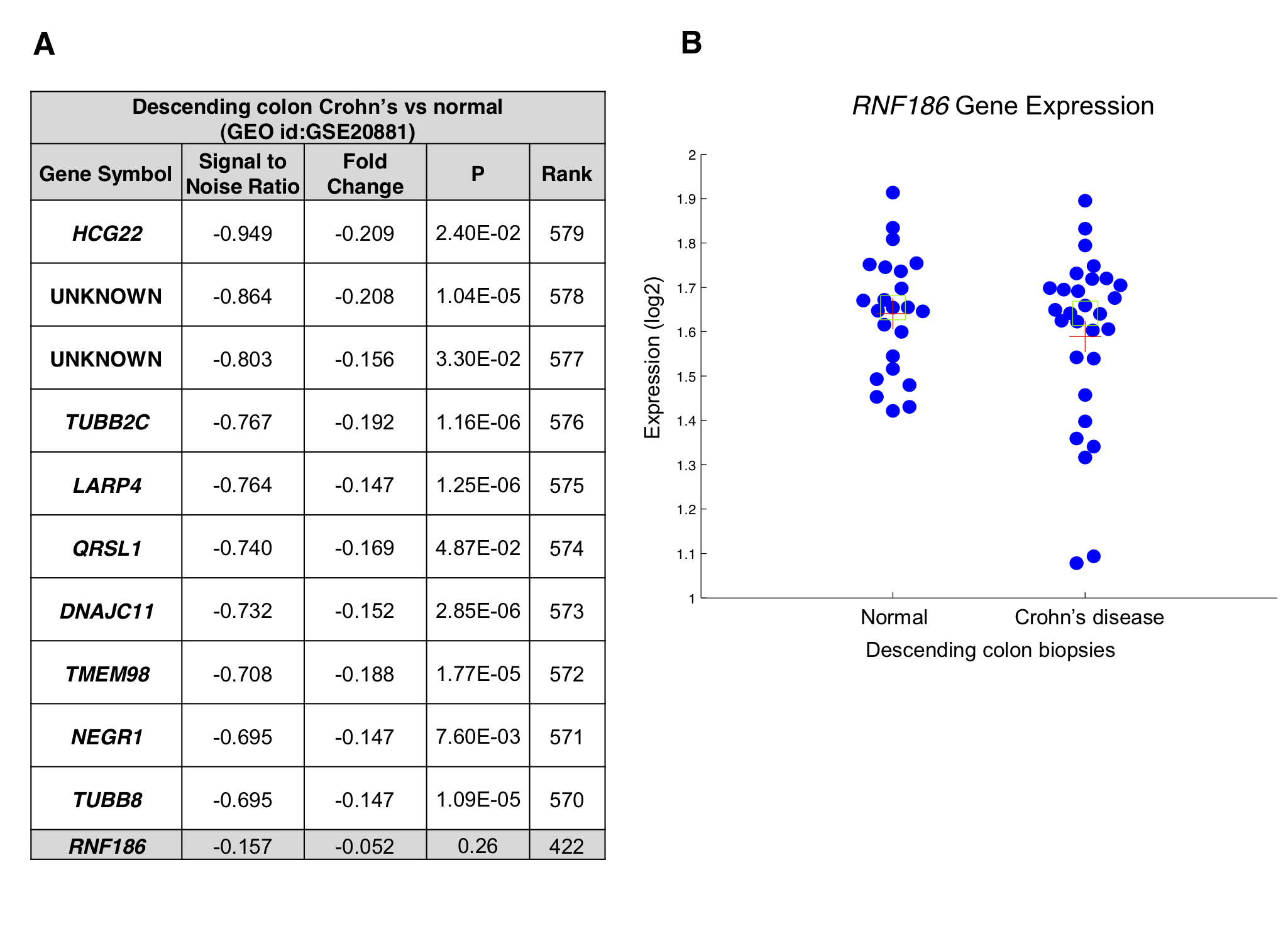

Supplement: Figure S7 — Comparative gene expression profiling of endoscopic biopsies taken at ileocolonoscopy from descending colon of Crohn's disease patients and healthy control donors. (A) Table of gene expression fold change statistics from transcriptional profiling of endoscopic biopsies taken at ileocolonoscopy from descending colon of Crohn's disease patients and healthy control donors (GSE20881). Only top 10 genes and RNF186 are shown. The rank column refers to the rank of the gene for signal to noise ratio in the specific study (579 significant genes ranked). (B) Plot of RNF186 gene expression in samples from descending colon biopsies of normal subjects and Crohn's disease patients. The squares and crosses represent median and mean respectively. (TIFF) [file pgen.1003723.s007.tiff]

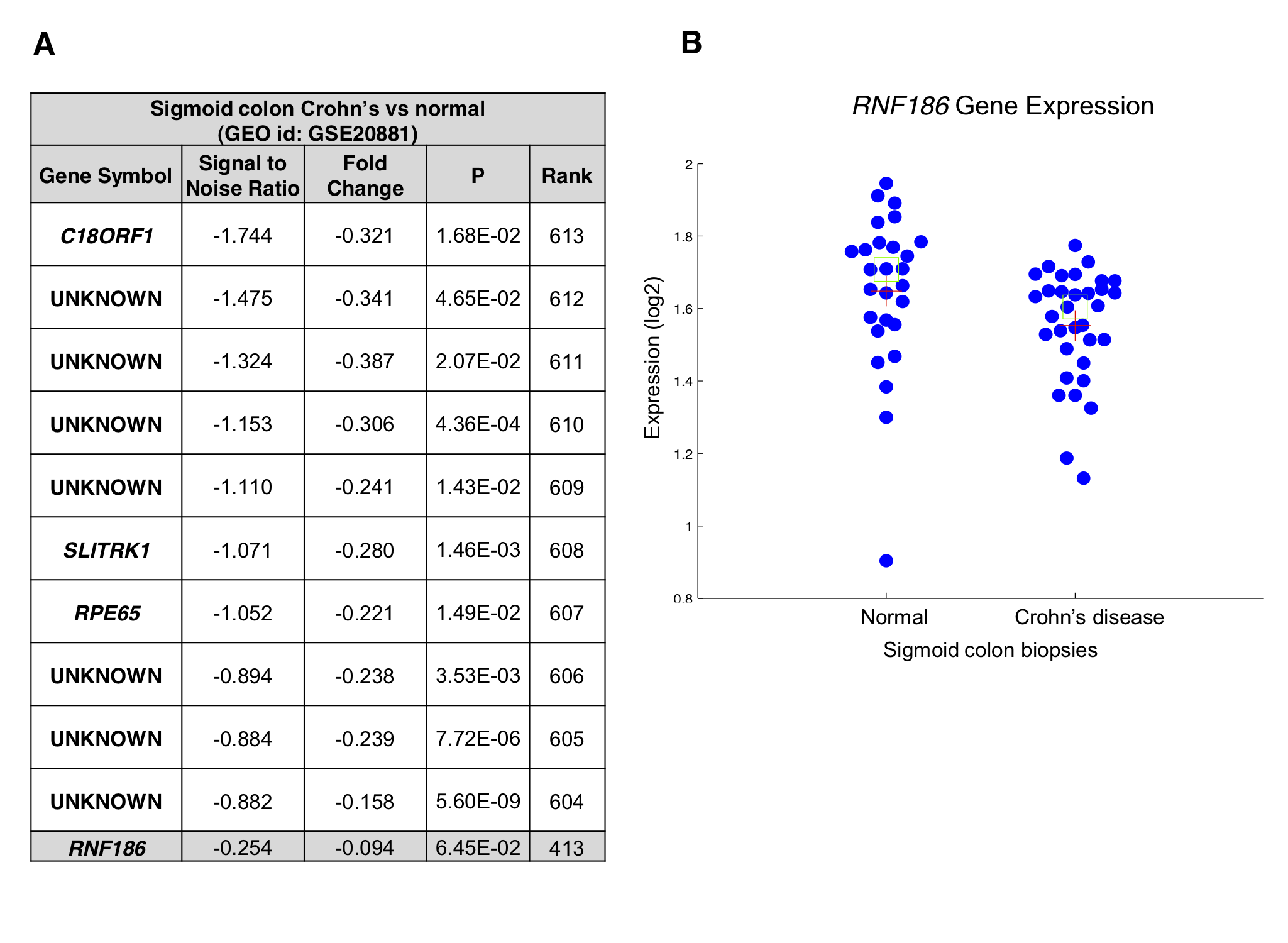

Supplement: Figure S8 — Comparative gene expression profiling of endoscopic biopsies taken at ileocolonoscopy from sigmoid colon of Crohn's disease patients and healthy control donors. (A) Table of gene expression fold change statistics from transcriptional profiling of endoscopic biopsies taken at ileocolonoscopy from sigmoid colon of Crohn's disease patients and healthy control donors (GSE20881). Only top 10 genes and RNF186 are shown. The rank column refers to the rank of the gene for signal to noise ratio in the specific study (613 significant genes ranked). (B) Plot of RNF186 gene expression in samples from sigmoid colon biopsies of normal subjects and Crohn's disease patients. The squares and crosses represent median and mean respectively. (TIFF) [file pgen.1003723.s008.tiff]

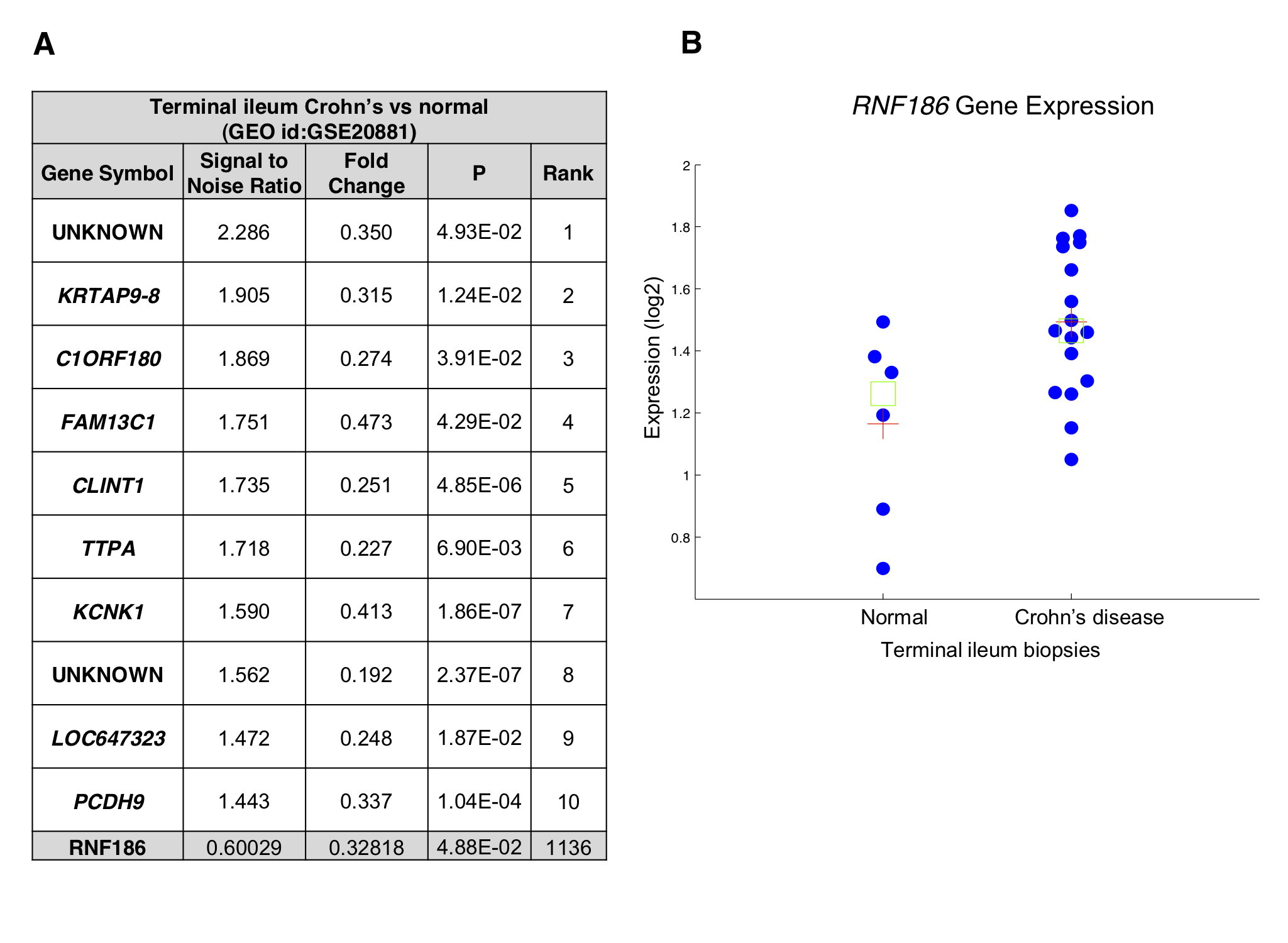

Supplement: Figure S9 — Comparative gene expression profiling of endoscopic biopsies taken at ileocolonoscopy from terminal ileum of Crohn's disease patients and healthy control donors. (A) Table of gene expression fold change statistics from transcriptional profiling of endoscopic biopsies taken at ileocolonoscopy from terminal ileum of Crohn's disease patients and healthy control donors (GSE20881). Only top 10 genes and RNF186 are shown. The rank column refers to the rank of the gene for signal to noise ratio in the specific study (2608 significant genes ranked). (B) Plot of RNF186 gene expression in samples from terminal ileum biopsies of normal subjects and Crohn's disease patients. The squares and crosses represent median and mean respectively. (TIFF) [file pgen.1003723.s009.tiff]

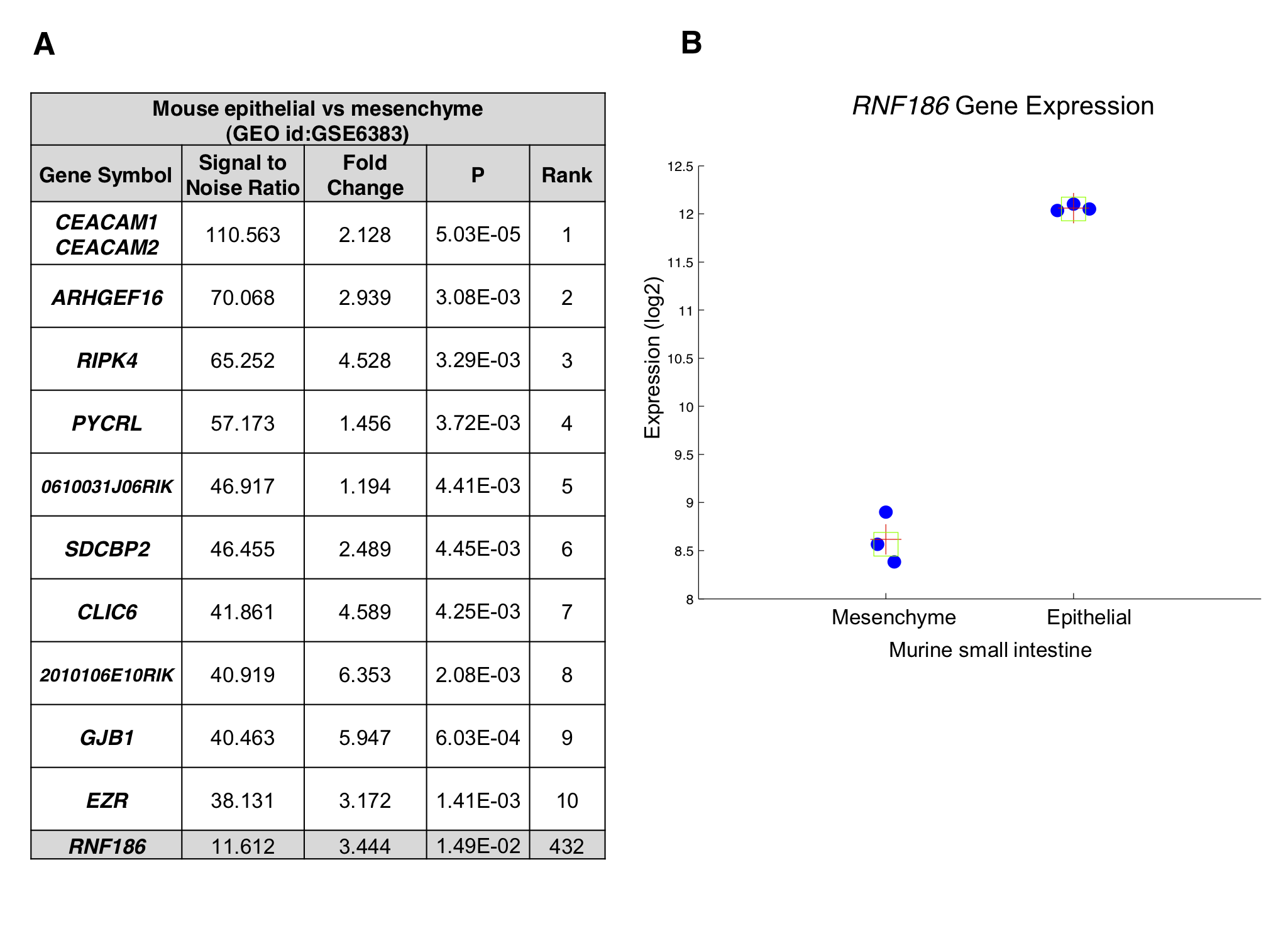

Supplement: Figure S10 — Comparative gene expression profiling of murine small intestinal epithelium and mesenchyme. A) Table of gene expression fold change statistics from transcriptional profiling of murine small intestinal epithelium and mesenchyme (GSE6383). Only top 10 genes and RNF186 are shown. The rank column refers to the rank of the gene for signal to noise ratio in the specific study (7239 significant genes ranked). (B) Plot of RNF186 gene expression in samples from murine small intestinal epithelium and mesenchyme. The squares and crosses represent median and mean respectively. (TIFF) [file pgen.1003723.s010.tiff]

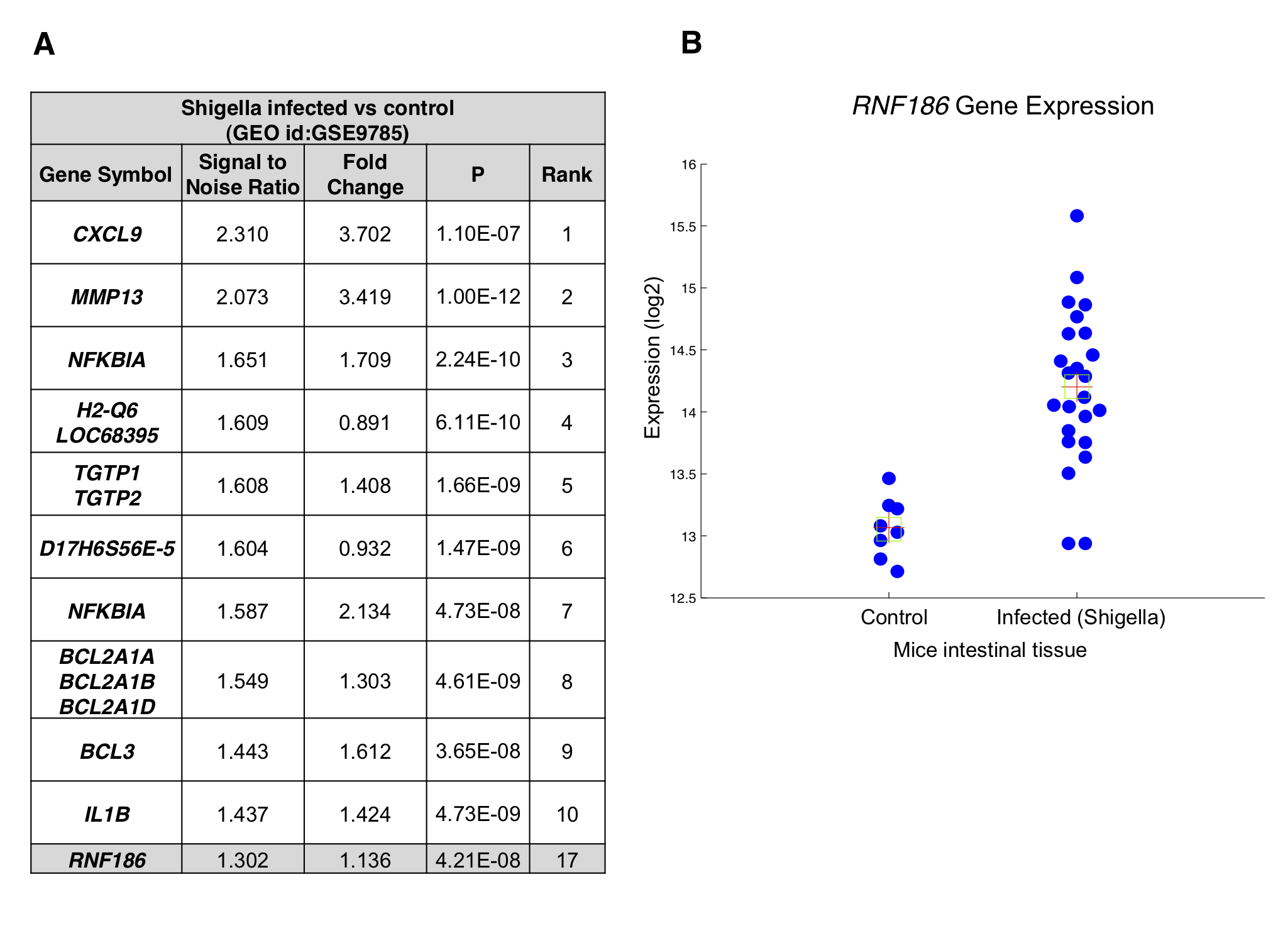

Supplement: Figure S11 — Comparative gene expression profiling of intestinal tissues of 4-day- or 7-day-old mice infected or not with invasive or non-invasive shigella. (A) Table of gene expression fold change statistics from transcriptional profiling of intestinal tissues of 4-day- or 7-day-old mice infected or not with invasive (INV+) or non-invasive (INV−) (GSE9785). Only top 10 genes and RNF186 are shown. The rank column refers to the rank of the gene for signal to noise ratio in the specific study (2258 significant genes ranked). (B) Plot of RNF186 gene expression in mice intestinal tissue infected with shigella and control samples. The squares and crosses represent median and mean respectively. (TIFF) [file pgen.1003723.s011.tiff]

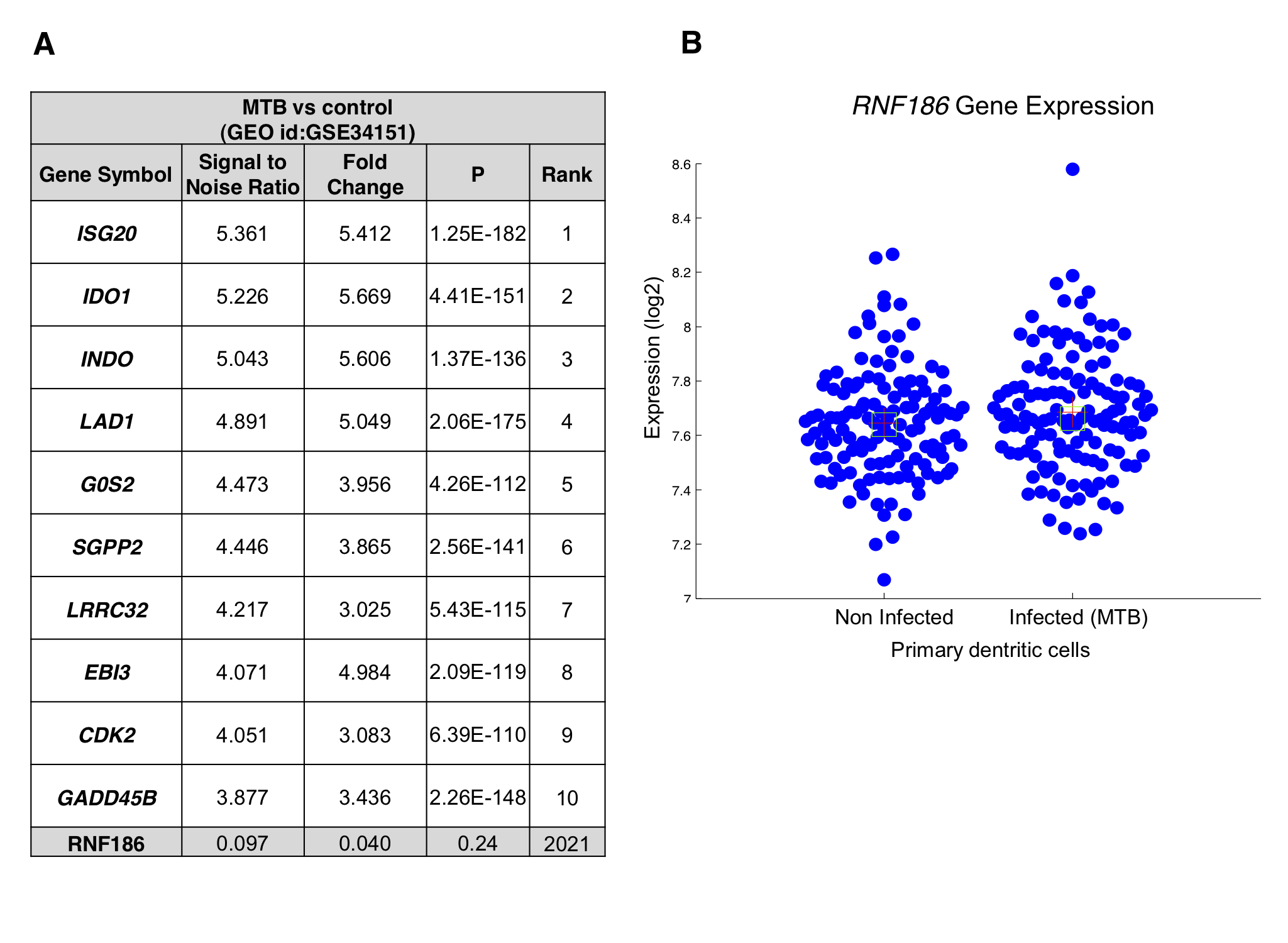

Supplement: Figure S12 — Comparative gene expression profiling in primary dendritic cells from 65 individuals, before and after infection with MTB. (A) Table of gene expression fold change statistics from transcriptional profiles in primary dendritic cells from 65 individuals, before and after infection with MTB (GSE34151). Only top 10 genes and RNF186 are shown. The rank column refers to the rank of the gene for signal to noise ratio in the specific study (4279 significant genes ranked). (B) Plot of RNF186 gene expression in primary dendritic cells from 65 individuals, before and after infection with MTB. The squares and crosses represent median and mean respectively. (TIFF) [file pgen.1003723.s012.tiff]

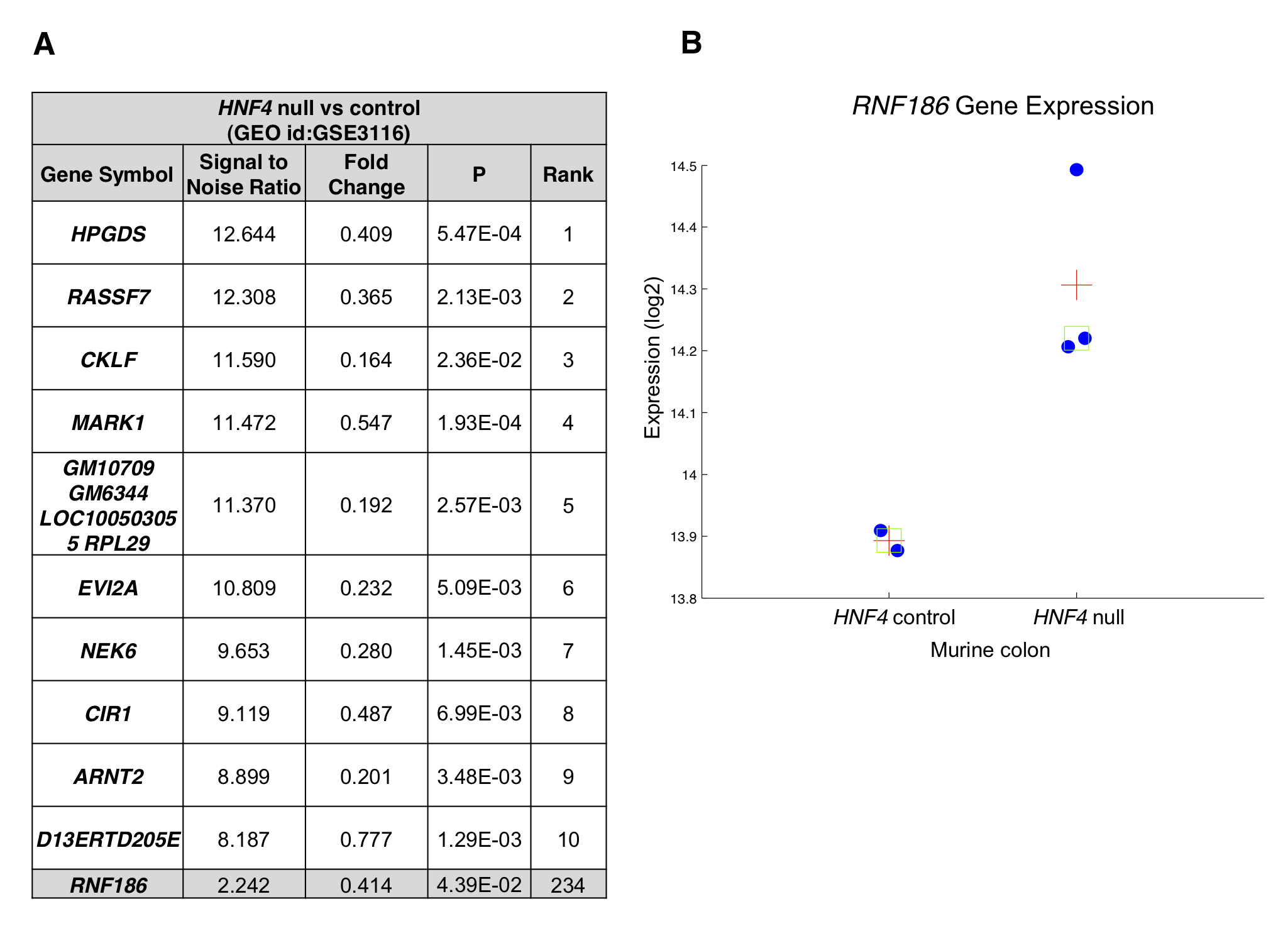

Supplement: Figure S13 — Comparative gene expression profiling in HNF4a mutant and control murine colons. (A) Table of gene expression fold change statistics from comparison of transcriptional profiles in HNF4a mutant and control murine colons (GSE3116). Only top 10 genes and RNF186 are shown. The rank column refers to the rank of the gene for signal to noise ratio in the specific study (895 significant genes ranked). (B) Plot of RNF186 gene expression in HNF4a mutant and control murine colons. The squares and crosses represent median and mean respectively. (TIFF) [file pgen.1003723.s013.tiff]

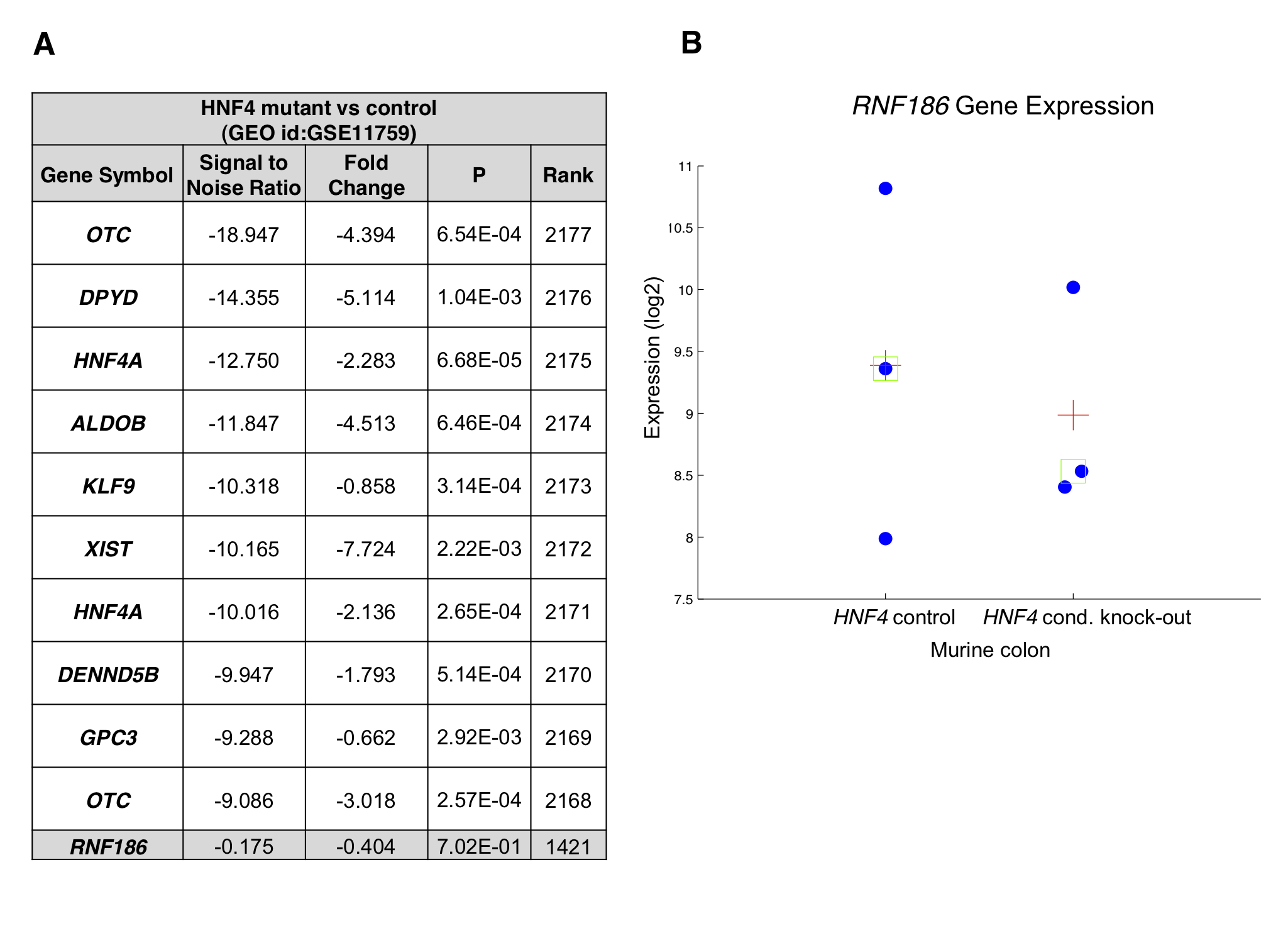

Supplement: Figure S14 — Comparative gene expression profiling in mouse epithelial colons with or without conditional knock out of HNF4. (A) Table of gene expression fold change statistics from comparison of transcriptional profiles in mouse epithelial colons with or without conditional knock out of HNF4 (GSE11759). Only top 10 genes and RNF186 are shown. The rank column refers to the rank of the gene for signal to noise ratio in the specific study (2177 significant genes ranked). (B) Plot of RNF186 gene expression in HNF4a conditional knock out and control murine colons. The squares and crosses represent median and mean respectively. (TIFF) [file pgen.1003723.s014.tiff]
